# Testis-specific actin-like 7A (ACTL7A) is an indispensable protein for subacrosomal-associated F-actin formation, acrosomal anchoring, and male fertility

**DOI:** 10.1093/molehr/gaad005

**Published:** 2023-02-03

**Authors:** P Ferrer, S Upadhyay, M Ikawa, T M Clement

**Affiliations:** Interdisciplinary Faculty of Toxicology Program, Texas A&M University, College Station, TX, USA; Department of Veterinary Physiology and Pharmacology, School of Veterinary Medicine and Biomedical Sciences, Texas A&M University, College Station, TX, USA; Department of Veterinary Physiology and Pharmacology, School of Veterinary Medicine and Biomedical Sciences, Texas A&M University, College Station, TX, USA; Department of Experimental Genome Research, Research Institute for Microbial Diseases, Osaka University, Suita, Osaka, Japan; Interdisciplinary Faculty of Toxicology Program, Texas A&M University, College Station, TX, USA; Department of Veterinary Physiology and Pharmacology, School of Veterinary Medicine and Biomedical Sciences, Texas A&M University, College Station, TX, USA

**Keywords:** *ACTL7A*, male fertility, infertility, acroplaxome, actin cytoskeleton, acrosome

## Abstract

Formation of the acrosome during spermiogenesis is an essential process for creating fertilization-competent sperm. Of the numerous aspects required for acrosome biogenesis, adherence of the acrosomal outer membrane to the nuclear surface is mediated by the subacrosomal perinuclear theca. However, the cellular dynamics and congruent functions pertaining to these acrosomal anchoring factors are not well understood despite many of them being implicated as potential causes for human male infertility. Actin-like 7A (ACTL7A) is one such factor for which deleterious polymorphisms have recently been shown to cause human male infertility. It is thought that acrosomal attachment is coordinated by cytoskeletal associations between the acrosome and nucleus via the acroplaxome. To further illuminate the mechanistic underpinnings of ACTL7A for essential acrosome associations, in this study, we investigated its dynamic localization in the developing germline, molecular associations with other cytoskeletal components, and the cellular consequences of ablation. Our intracellular localization data show ACTL7A to be dynamically present within the nucleus and subacrosomal space and later associated with postacrosomal regions of developing spermatids. Through the generation of an *Actl7a* knock-out mouse model, we consistently observed disruption of acrosomal biogenesis with abnormal migration of the acrosomal granule and peeling acrosomes during spermatid elongation. Significantly, we found a complete loss of subacrosomal filamentous actin (F-actin) structures in knock-out spermatids suggesting a regulatory role for subacrosomal F-actin. Considering our reported data together with existing literature, we propose a mechanistic model explaining the essential role of ACTL7A for acroplaxome-associated F-actin, acrosomal attachment integrity, and male fertility.

## Introduction

Spermatogenesis is a robust and dynamic process of cellular maturation where the basally located spermatogonia migrate luminally through the seminiferous epithelium and mature into highly specialized spermatozoa. This radical morphological transformation of the male germline is largely dependent on the support of the adjacent Sertoli cells, but also requires the formation of three unique cytoskeletal suprastructures intrinsic to the developing spermatids: (i) the flagellum, a multifarious structure responsible for the motility of sperm (Thorell, 1866; Kiebel and Mall, 1911; Lowndes, 1947; Fawcett and Phillips, 1969; Iwashita and Oura, 1980); (ii) the manchette, a tubulin-actin cuff, which facilitates nuclear reshaping (Russell *et al.*, 1991) and provides a microtubule highway for motor protein-directed active transport (Porter, 1973; Hayasaka *et al.*, 2008); and (iii) the perinuclear theca, a dense cytoskeletal foundation, which surrounds the spermatid’s nucleus and aids in the organization of other cytoskeletal assemblies like the manchette (Russell *et al.*, 1991; Müjica *et al.*, 2003) and the anchoring of organelles like the acrosome (Vogl, 1990). Ultimately, each of these cytoskeletal structures is anchored to the nuclear envelope in spermiogenesis. In addition, these regions of cytoskeletal adherence to the nuclear envelope can be compartmentalized into four distinct regions, each having its own unique protein composition and function: (i) the implantation fossa where the growing flagellum and its adjacent components first anchor themselves to the base of the nucleus (Fawcett and Phillips, 1969; Iwashita and Oura, 1980; Wu *et al.*, 2020); (ii) the postacrosomal sheath that fully develops during the later stages of spermiogenesis and is believed to aid in membrane fusion, oocyte activation (Kashir *et al.*, 2015) and cargo distribution from epididymosomes (Zhou *et al.*, 2019); (iii) the equatorial groove belt where both the acrosomal marginal ring and manchette are anchored (Kierszenbaum *et al.*, 2011); and (iv) a dense cytoskeletal scaffolding structure between the nuclear envelope and acrosome called the acroplaxome, which has been theorized to be responsible for acrosomal anchoring and shaping around the nucleus of spermatogenic cells (Kierszenbaum *et al.*, 2003). Before the term acroplaxome was coined, filamentous actins (F-actins) had previously been observed in the subacrosomal space of developing spermatids via electron microscopy (Vogl, 1990) but their overall structural arrangement was believed to be too undefined at the time in order to differentiate them as their own separate structure housed within this intracellular space. This particular distinction between the perinuclear subacrosomal space and acroplaxome was first made through the observation of distinct localization patterns of cytokeratin 5 (formerly known as Sak57 in mice) and β-actin to the subacrosomal space and the acrosomal marginal ring in spermatids (Kierszenbaum *et al.*, 2003). These initial observations coalesced into a proposed model in which there is an organizational directive on which proteins are localized to different regions of the subacrosomal space (rather than there just being an undifferentiable mesh of actin filaments throughout the region) to conjoin the nucleus and acrosome through an elegantly discreet cytoskeletal anchoring complex within the subacrosomal perinuclear theca; more broadly, the model adapted the use of the term acroplaxome when referring to this cytoskeletal structure.

From the conceptualization of the acroplaxome, many studies over the years have tried to make the distinction between the possible localization patterns of proteins found within the subacrosomal perinuclear theca region and, more specifically, localize them to the nuclear envelope, acroplaxome, subacrosomal space, and inner acrosomal membrane. Existing literature suggests that proteins such as DPY19L2 (Pierre *et al.*, 2012), FAM209 (Castaneda *et al.*, 2021), SPATA46 (Chen *et al.*, 2016), and LINC complexes (Göb *et al.*, 2010; Pierre *et al.*, 2012; Kmonickova *et al.*, 2020) are associated with the nuclear envelope below the subacrosomal space, while proteins like ZPBPs (Kanemori *et al.*, 2016; Chen *et al.*, 2021), SPACA1 (Fujihara *et al.*, 2012; Chen *et al.*, 2021), and IZUMO3 (Ellerman *et al.*, 2009; Inoue *et al.*, 2021) are found near the inner acrosomal membrane above the subacrosomal space. Yet, even though cytoskeleton-associated proteins like cytokeratin 5 (Kierszenbaum *et al.*, 2003), β-actin (Kierszenbaum *et al.*, 2003), CAPZA3 (Geyer *et al.*, 2009; Crapster *et al.*, 2020), MgcRabGAP (Lin *et al.*, 2010), Myosin Va (Kierszenbaum *et al.*, 2004), PFN 3 and 4 (Obermann *et al.*, 2005; Behnen *et al.*, 2009), PRAME L1 and X1 (Liu *et al.*, 2021), and other actin-related/binding proteins (ARPs and ABPs, respectively) are known to be in the subacrosomal space and are speculated to partake in the acroplaxome, the structural organization of these protein components within this intracellular compartment are largely unknown.

Over the past two decades, there has been an increasing focus on characterizing the effects of both protein polymorphisms and specific protein deficiencies on male fertility. This change in prioritization has yielded better insight into the spermatid-specific functionality of many proteins. The results of these studies are vast, but a small subset of them have reported a shared phenotypic quality: that of peeling acrosomes, resulting in severe subfertility to complete infertility. As a result, we have been able to differentiate between proteins that are present in the subacrosomal space and required for acrosomal anchoring or not, thus further pinpointing their specific roles in acrosome biogenesis and function. Most recently, the depicted phenotype of peeling acrosomes has been described in humans where coding point mutations in the actin-like 7A (*ACTL7A*) (Xin *et al.*, 2020) and *ACTL7C* (aka *ACTL9*) (Dai *et al.*, 2021) genes have been identified as pathogenic variants of significance in cases of complete human male infertility. Additionally, separate murine studies have described the same occurrence of acrosomal instability when knocking out protein kinase *Hipk4* (Crapster *et al.*, 2020), actin-related testis 1 (*Actrt1*) (Zhang *et al.*, 2022b), actin-binding protein profilin 3 (*Pfn3*) (Umer *et al.*, 2021), actin-like 7B (*Actl7b*) (Clement *et al.*, 2023), and *Actl7a* (Zhou* et al.*, 2022). Given the overlapping phenotype of these mutant mouse lines, the implication is that these proteins are essential for male fertility through their shared function in acroplaxome formation, regulation, stability, and conservation in mammals including humans. The general localization pattern and a handful of interacting partners are known for some of these proteins, but their relative mechanisms of action and how they affect F-actin’s formation and regulation within the acroplaxome are still largely unknown. We seek a better understanding of the roles of testis-specific and enriched proteins to increase our understanding of adapted cytoskeletal regulation in spermiogenesis and the mechanistic understanding of male infertility.

In this study, we investigate the importance of ACTL7A expression for male fertility. ACTL7A is a member of the ARP family of proteins that share a conserved actin domain (Goodson and Hawse, 2002). Ubiquitously expressed ARPs play essential roles in cytoskeletal dynamics, motor transport, and chromatin remodeling. ACTL7A is a testis-specific expressed ARP containing the conserved actin domain and a unique intrinsically disordered N-terminal domain of ∼70 amino acids (aa), which is conserved in mammals and has been shown to bind the LIM1-2 domains of Testin (Boëda *et al.*, 2011). We sought to better understand the roles of ACTL7A in spermatogenesis, acroplaxome biogenesis/function, and actin cytoskeletal regulation in spermiogenesis by generating an *Actl7a* knock-out (KO, *Actl7a^−/−^*) mouse model. Herein, we elucidate the spatiotemporal molecular association of ACTL7A with specific intracellular regions, identify actin-related protein 2 (ARP2), dynactin subunit 1 (DCTN1), myosin 6 (MYO6), and profilin 4 (PFN4) as interacting proteins, and show that ACTL7A is specifically required for the presence of F-actin in the subacrosomal perinuclear theca.

## Materials and methods

### Ethics statement

All experiments involving animals were approved by the Institutional Animal Care and Use Committees of Texas A&M University (IACUC #2018-0104) and/or Osaka University (RIMD-AP #R03-01-0.) and were conducted in compliance with the guidelines and regulations for animal experimentation of these institutions.

### Generation of *Actl7a* KO mice

Mice were generated at Osaka University using CRISPR/Cas9 following previously reported procedures (Abbasi *et al.*, 2018). The crRNA target sequences to generate gRNAs were: 5′ crRNA: CAGAAAGCTCTCGATCCTGC/TGG and 3′ crRNA: GTTCCTCGTACTCCAGGCGA/TGG. Electroporation of guide RNA complexes into one-cell C57BL6/n embryos and transfer of embryos into pseudopregnant ICR female mice to produce F0 heterozygous offspring was performed as previously described (Abbasi *et al.*, 2018). This generated mice with a 1306 + 2 bp deletion of the single exon coding region for *Actl7b* encompassing 23 bp upstream of the translational start site codon to 49 bp upstream of the translational stop codon verified by Sanger sequencing. Subsequently, mice were acclimated to a 12-h light/12-h dark cycle at Texas A&M University and given food and water *ad libitum*. Heterozygotes underwent serial mating to produce homozygous mutant *Actl7b^−/−^* offspring. For fertility trials, males of at least 8 weeks of age were paired with wild-type (WT) C57BL6/n females 10–16 weeks of age for 4 months. Monitoring of females for pregnancy and litters continued through another gestational length (4 months + 21 days) to record outcomes of the four breeding months. Litter dates, pups, and gender ratios were recorded. Pups per litter, litters per month, and pups per month were calculated for each sire to compare fertility.

### PCR genotyping of subsequent generations

Genomic DNA was isolated from tail biopsies using the Purlink Genomic DNA mini kit (#K1820-02, Invitrogen, Waltham, MA, USA). The forward genotyping primer (F1-WT and KO) was the same for the WT and KO alleles: 5′-ACCAGATAAGGGTGGGGTTC-3′. The WT reverse primer (R1-WT) was 5′-GTGCCCCACAAAGGTCTCT-3′, generating a 675 bp product. The KO reverse primer (R2-KO) was 5′-TTGCTCCCGTGAGAGACTTT-3′, producing a 596 bp product. The PCR amplifications were performed with Phusion Plus PCR master mix (#F631S, ThermoFisher Scientific, Waltham, MA, USA) with amplification conditions being initial denaturation at 98°C (30 s), then 30 cycles of denaturation 98°C (10 s), annealing 57°C (10 s), extension 72°C (15 s), and a final extension of 72°C (5 min).

### Sperm count and motility

Murine sperm was collected by making several cuts to mince the cauda epididymis with iridectomy scissors in 1 ml of 37°C human tubal fluid (HTF) media (#MR-070-D, Sigma-Aldrich, Saint Louis, MO, USA) supplemented with 3 mg/ml albumax (#11020021, Gibco, Billings, MT, USA) and then allowing the sperm to naturally propagate outwards from the tissue for 15 min (herein referred to as T0 sperm). Then, 200 µl of T0 sperm was added to 800 µl of fresh bovine serum albumin (BSA)-rich HTF and further incubated for 90 min at 37°C (herein referred to as T90 sperm). Sperm counts were performed in a standard Neubauer chamber from a 1:20 dilution of T0 sperm to DI water. A subset of both T0 and T90 sperm were fixed for 30 min with 4% paraformaldehyde (PFA) for capacitation assessments. Another subset of sperm from each timepoint were subjected to computer-assisted sperm analysis (CASA) as previously described (Goodson *et al.*, 2011). CASA was performed on a Hamilton Thorne IVOS II system (Hamilton Thorne Biosciences, Beverly, MA, USA) with 4× negative phase contrast when their respective incubation times reached their completion. Sperm were diluted to 1 × 10^6^ sperm/ml in HTF +albumax and 30 µl were loaded onto prewarmed 100 µm deep Leja counting chamber slides (Leja Products, B.V., Nieuw-Vennep, The Netherlands) for analysis. A minimum of 10 fields and 300 sperm were measured for each sample. Preset values for analysis consisted of the following: exposure = 16 ms, gain = 500, integrate enabled = false, integrate time = 500 ms, head size max = 80 µm^2^, head size minimum = 7 µm^2^, static tail filter = false, tail brightness = 68, tail brightness auto offset = 10, tail min brightness mode = auto first frame, illumination type = visible, intensity = 2580, max photometer = 60, min photometer = 55, progressive straightness (STR) = 50%, progressive VAP = 50 µm/s, slow VAP = 10 µm/s, slow VSL = 10 µm/s, static VAP = 5 µm/s, static VSL = 5 µm/s, 90 frames acquired at 60 f/s.

### TUNEL assays

PFA-fixed sperm were settled onto poly-l-lysine-coated slides overnight and stained with the Click-iT Plus TUNEL assay kit (#C1067, Invitrogen, Waltham, MA, USA) according to the manufacturer’s protocol. Sperm were counterstained with DAPI and mounted with Prolong Gold Antifade (Invitrogen, Waltham, MA, USA). Assessments were made on a Leica DMi8 microscope scoring a minimum of 200 sperm for each of five biological replicates.

### CMA3 chromatin assays

Sperm samples were settled onto poly-l-lysine-coated glass slides overnight. The sperm were permeabilized with 0.5% NP40 in PBS for 2 min and stained with 0.25 mg/ml chromomycin A3 (#C2659, Millipore Sigma, Burlington, MA, USA) in 10 mM MgCl_2_, 164 mM Na_2_HPO_4_, and 17 mM citric acid, pH 7.0 for 20 min. Samples were counter stained with Hoechst 33342 (#14533, Millipore Sigma) and mounted with Prolong Gold Antifade (#P36930, ThermoFisher Scientific, Waltham, MA, USA). A minimum of 200 sperm were scored for each of five biological replicates.

### Histology

The tunica albuginea of dissected murine testis was opened with a ∼1 mm linear incision with iridectomy scissors and fixed in a 4% solution of PFA or Bouin’s fixative at 4°C for 24 h. After the first pass of fixation, the testes were hemisected and fixed for an additional 48 h under the same conditions before washing and paraffin embedding according to the standard procedures. Embedded tissues were sectioned to a thickness of 5 µm, mounted on statically charged slides, dewaxed, and rehydrated. Sequentially, the prepared slides were stained with hematoxylin (#S211-16OZ, Poly Scientific, Bay Shore, NY, USA) and periodic acid Schiff’s reagents (#1090341000, Millipore, Burlington, MA, USA) according to the standard protocols, and then imaged at 63× using a Leica DMi8 S Platform inverted microscope system. Lastly, for the visualization of acrosomal granule displacements in *Actl7a*^−/−^ spermatids, we utilized the innate fluorescence of the PAS stain in Bouin’s fixed testis using a Y5/CY5 red filter cube with an excitation filter of 620/60 nm, a dichromatic mirror for 660 nm, and a suppression filter of 700/75 nm.

### Testicular cell singularization

Adult murine testes were detunicated and suspended in 10 ml of Krebs-Ringer bicarbonate buffer (EKRB) (Bellve *et al.*, 1977). Seminiferous tubules were then enzymatically digested by the addition of 2.5 mg of collagenase IV (#SCR103, Sigma-Aldrich, Saint Louis, MO, USA) for 15 min in a shaking incubator at 37°C, 5% CO_2_, and 150 rpm, followed by a digestion with 5 mg of trypsin III from bovine pancreas (#T9201, Sigma, Saint Louis, MO, USA) with 1 mg of DNase I (#D4527, Sigma, Saint Louis, MO, USA) in EKRB for 30 min. The digested tissue was then homogenized by gentle pipetting for 4 min followed by the addition of 5 mg of trypsin inhibitor (#T9003, Sigma-Aldrich, Saint Louis, MO, USA) at the completion of this process. Lastly, the singularized cell mixture was filtered through a 70 µm nylon mesh (#352350, Falcon, Cary, NC, USA), centrifuged at 250×*g* for 10 min, and fixed with 4% PFA for 30–60 min.

### Protein extraction and coimmunoprecipitation

Independently of one another, both murine testis and YFP-6His-ACTL7A-transfected HEK293F cells were frozen in liquid nitrogen and pulverized using a BioPulverizer (#59014N, Biospec Products, Bartlesville, OK, USA). The cryogenic lysate powders were then resuspended in a low salt buffer containing 10 mM Tris-HCl, 205 µM CaCl_2_, and 100 mM KCl at a pH of 7.8 before being further processed by a Dounce homogenizer. The homogenized lysate mixtures were then centrifuged at 10 000×*g* for 15 min at 4°C before collecting the supernatant (soluble fraction) for further use. The soluble fractions of both testis lysate and the transfected HEK cell lysates were then coincubated for 1 h under nutation at room temperature. Agarose-conjugated GFP affinity nanobody beads (#GTA, Chromotek, Planegg, Bayern, Germany) were then added to the lysate mixture and incubated overnight under nutation at 4°C. Finally, the beads were washed with three changes of low salt buffer for 10 min before adding 100 mM glycine at a pH of 2.8 to elute out ACTL7A and any additional protein constituents which may have copurified.

### SDS-PAGE western blot

All protein samples for western blot analysis were diluted at a 1:1 ratio with a solution of 2× Laemmli sample buffer (#1610737EDU, Bio-Rad, Hercules, CA, USA) with a 5% content of anhydrous 2-mercaptoethanol (#1610710XTU, Bio-Rad, Hercules, CA, USA) and boiled for 15 min. For each sample, 35 µl was loaded per well in 10–20%, Tris-Glycine, 1.0 mm denaturing gels (#XP10200BOX, Invitrogen, Waltham, MA, USA). After the protein contents of all samples were separated by gel electrophoresis, they were transferred to a polyvinylidene difluoride membrane (#1620174, Bio-Rad, Hercules, CA, USA), washed with TBST (0.1% Tween-20), blocked overnight at 4°C with a 5% dry nonfat milk solution, and subsequently incubated with primary antibodies at a 1:5000 dilution factor for a minimum of 2 h to overnight at 4°C with a 5% milk content. Corresponding anti-rabbit or anti-goat secondary horseradish peroxidase-conjugated antibodies were then added at a 1:10 000 dilution factor in TBST. Western blot signals specificity and intensity were captured using the Bio-Rad ChemiDoc XRS Imaging System and analyzed via the ImageLabs software. A comprehensive list of antibodies used can be found in Supplementary Table SI.

### Silver staining

All protein samples for silver stain analysis were diluted at a 1:1 ratio with a solution of 2× Laemmli sample buffer (#1610737EDU, Bio-Rad, Hercules, CA, USA) with a 5% content of anhydrous 2-mercaptoethanol (#1610710XTU, Bio-Rad, Hercules, CA, USA) and boiled for 15 min. For each sample, 35 µl was loaded per well in 10–20%, Tris-Glycine, 1.0 mm denaturing gels (#XP10200BOX, Invitrogen, Waltham, MA, USA). After all protein products were separated by gel electrophoresis, the Tris-Glycine gels were fixed with a solution of 30% EtOH and 10% acetic acid for 30 min. Following fixation, the gels were washed twice with 10% EtOH for 5 minutes and then with ultrapure water before proceeding with staining with the ThermoFisher Scientific™ Pierce™ Silver stain kit (#PI24612, ThermoFisher Scientific, Waltham, MA, USA) as per the provided manufacturer’s guidelines. Protein band signals from the silver stain were captured using the Bio-Rad ChemiDoc XRS Imaging System and analyzed via the ImageLabs software.

### Immunofluorescence

Fixed sperm and singularized germ cells or HEK293F cells were settled on poly-l-lysine-coated slides overnight and permeabilized with phosphate buffered saline with 1% Triton X-100 (PBST) for 15 min. Slides were washed with PBS and then blocked with 10% horse serum for 30 min before proceeding with antibody staining. Slides were stained with rabbit and/or mouse primary antibodies at a 1:200 dilution in PBS for 2 h, sequentially washed with three changes of PBS for 5 min, and then incubated with anti-mouse and/or anti-rabbit Alexa-conjugated secondary antibodies for 1 h. Peanut agglutinin (PNA) and/or phalloidin were used at a 1:400 dilution for 1 h, coincident with secondary antibodies when colabeling. DAPI was used to counter-stain nuclei at a dilution of 1:500 for 3 min. Slides were then mounted with Aqua-Polymount (#18606, Polysciences, Warrington, PA, USA) and imaged at 63× using a Leica Dmi8 S Platform (LAS X software V5.0.2) inverted microscope system for widefield microscopy or a Zeiss LSM 780 multiphoton microscope (ZEN Blue software V3.4) for confocal microscopy. Images of germ cells and HEK293F cells were assessed for localization patterns relative to cell type and (for germ cells) developmental step.

### Spermatid staging, scoring of ACTL7A stage-specific localization, and heatmap generation

Individualized WT germ cells from three separate mice were fluorescently labeled with DAPI, PNA, and anti-ACTL7A IgG. Individual spermatids were then analyzed using a Leica Dmi8 S Platform inverted widefield microscope at 100× magnification and categorized into their respective spermatogenic steps (15–60 cells per step, per mouse) based on previously reported morphological guidelines (Russell *et al.*, 1993). In essence, round spermatids were staged based on acrosomal marginal edge to nuclear center angles, subacrosomal nuclear concavity, acrosomal outer membrane to cell membrane distance, nuclear position in the cytoplasm, and location of the Golgi apparatus relative to the acrosome and nucleus (Russell *et al.*, 1993). Elongating and condensing spermatids were staged based on nuclear dimensions (total length and width), topological morphology of the caudal part of the nucleus surrounding the implantation fossa, nuclear symmetry, perforatorium development, acrosomal morphology, and flagellar development (Russell *et al.*, 1993). Given the apparent defects in the perforatorium and acrosomes present in elongating and condensing *Actl7a*^−/−^ spermatids, we restrictively used nuclear width, topological morphology of the caudal part of the nucleus surrounding the implantation fossa, and flagellar development to approximate their stage of development. Regarding WT spermatids, it is important to note that each cell was individually categorized using the differential interference contrast (DIC), DAPI, and PNA channels exclusively, prior to observing their respective ACTL7A stain, in order to prevent selection bias. After each observed germ cell was categorized based on their developmental step, the intracellular presence of ACTL7A was recorded at multiple planes of focus for the following intracellular regions: the cytoplasm, nucleus, acroplaxome, marginal ring, neck, and postacrosomal space. To quantify the expression and localization patterns over spermatid development, the percentage of germ cells per step with ACTL7A localized at each of the aforementioned intracellular spaces (scored as present or absent in each structure rather than scored as expression intensity) was arranged as a spatiotemporal expression heatmap of the protein using the GraphPad Prism V9 software for better visualization.

### Plasmid expression and HEK293F transfection

Our designed YFP-6His-tagged human *ACTL7A* vector was constructed using the Gateway cloning system (Invitrogen, Waltham, MA, USA). The *ACTL7A* human gene sequence with an N-terminal TEV site and coded 6His tag was cloned into the Gateway plasmid pENTR vector using the pENTR d-Topo cloning kit (Invitrogen) using the manufacturer’s protocol. The TEV-6His-*ACTL7A* insert was then transferred from the entry clone to the pcDNA6.2/N-YFP-DEST vector using the LR clonase reaction (Gateway^®^ LR clonase™ II Enzyme Mix, Invitrogen, Waltham, MA, USA) to create the pcDNA6.2N-YFP-6His-*ACTL7A* plasmid (Supplementary Fig. S1). A complementary control vector lacking only the *ACTL7A* coding sequence was also generated. The sequence fidelity of the clones was verified by DNA sequencing. The plasmid DNA was propagated in *Escherichia coli* and isolated using the Endo-free plasmid maxi kit (#12162, Qiagen, Valencia, CA, USA).

HEK293F cells (#R79007, ThermoFisher Scientific, Waltham, MA, USA) were passaged and transfected with purified pcDNA6.2N-YFP-6His-*ACTL7A* plasmid using the Freestyle 293 Expression System and the recommended reagents and guidelines provided by the supplier. In short, HEK293F cells were cultured in suspension using Freestyle™ 293 Expression Medium (#12338026, ThermoFisher Scientific, Waltham, MA, USA) at 37°C and 5% CO_2_ and passaged accordingly when they reached a cell population density between 1 × 10^6^ and 3 × 10^6^ cells per ml of media with a cell viability above 94%. After approximately three passages, the HEK293F cell culture was transfected with a ratio of 1 part pcDNA6.2N plasmid construct to three parts Lipofectamine™ 3000 (#L3000015, ThermoFisher Scientific, Waltham, MA, USA). After transfection, cells were cultured for 14–18 h before harvesting for protein isolation as mentioned above or fixed with 4% PFA for 1 h and stored at 4°C for follow-up immunofluorescence assessments.

### Statistics

All data pertaining to changes in sperm motility parameters, tissue weights, fertility trials, and prevalence of TUNEL and CMA3+ positive sperm were statistically determined through one-way ANOVA analyses performed in GraphPad Prism version 9.0. All applicable statistical analyses were performed at a 95% CI.

## Results

### *Actl7a* KO mice are infertile and exhibit consistent head and acrosomal morphological defects in sperm

**Figure 1.**
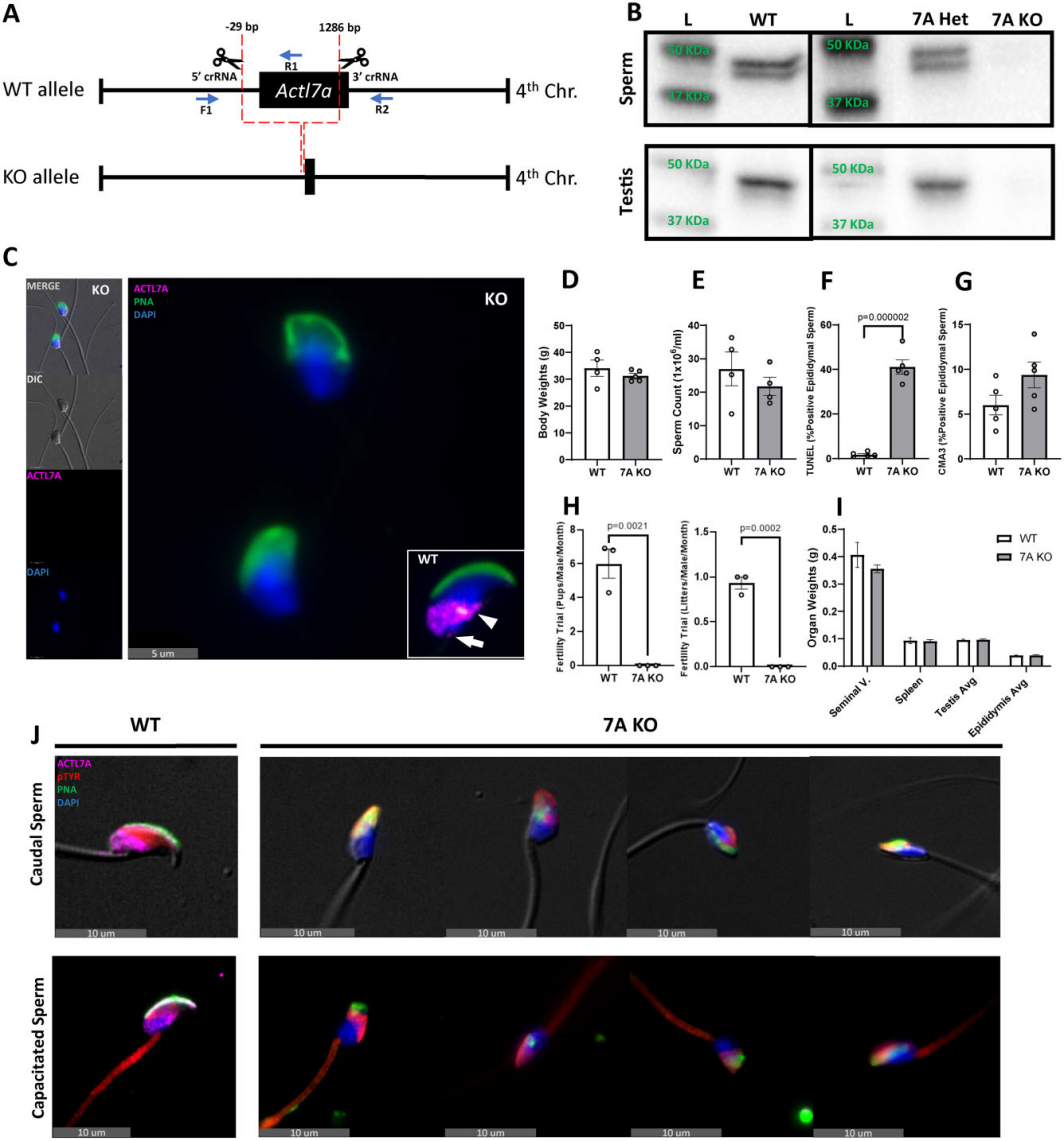
***Actl7a* KO mice have abnormal sperm morphology and are infertile.** (**A**) Diagram illustrating the crRNA sites for creation of *Actl7a* KO mice using the CRISPR/Cas9 system and genotyping primers F1, R1, and R2. (**B**) *Actl7a* KO validation via western blot in testis and sperm. (**C**) Comparison of immunofluorescent labeled sperm between KO and WT mice. *Actl7a* KO sperm is devoid of ACTL7A staining and consistently exhibits abnormal head morphology. WT sperm ACTL7A localizes in the postacrosomal sheath region (arrowhead) and connecting piece region (arrow). (**D**–**I**) Comparison of different health and fertility metrics between *Actl7a* KO and WT mice. Statistically significant differences are indicated for fertility and sperm TUNEL assays. (**J**) Comparison between KO and WT sperm pre- and postcapacitation, showing changes in flagellar localized immunofluorescent phosphorylated tyrosine (pTYR) staining. All error bars are representative of the SEM. ACTL7A, actin-like 7A; F, forward; R, reverse; KO, knock-out; WT, wild type.

To better understand the roles of ACTL7A and its effect on spermatogenesis, we generated *Actl7a* KO mice using the CRISPR/Cas9 system to delete the coding sequence of the intronless *Actl7a* gene (Fig. 1A). Genotypes were validated by PCR and sequencing of the genomic amplification product (not shown) and through protein assessments by western blot analysis of both sperm and testis lysates, as well as indirect immunofluorescent microscopy (Fig. 1B and Supplementary Figs S2A and B and S3). A single ACTL7A band in testis, and two sperm-specific ACTL7A bands observed in WT and heterozygous testis lysates were not detectable in the KO (Fig. 1B). ACTL7A was also not detected via indirect immunofluorescent microscopy in KO sperm or singularized germ cells (Fig. 1C and Supplementary Fig. S2A and B). When *Actl7a* KO male mice were paired with WT female mice, complete infertility was observed (Fig. 1H). Non-testis-related phenotypes were not observed. The body weights (Fig. 1D), spleen weights (Fig. 1I), and weights of hormone-responsive seminal vesicles (Fig. 1I) were all normal. Testis and epididymal weights (Fig. 1I) and sperm counts (Fig. 1E) were also comparable between *Actl7a^−/−^* KO and WT (*Actl7a^+/+^*) males indicating that sperm production rates were unaffected. However, a difference in sperm morphology was observed. In WT mouse sperm, a hook-shaped structure forms at the apex of the tapered sperm head called the perforatorium. Initial morphological characterization of *Actl7a* KO sperm showed a consistent loss of both the perforatorium and the iconic crescent shape of the acrosome found in non-acrosome reacted sperm (Fig. 1C). *Actl7a* KO mice were observed to have a significant increase in TUNEL positive sperm (Fig. 1F and Supplementary Fig. S4A and B), indicating that DNA damage, typically considered to imply cell death, was increased. However, CMA3 staining as an indicator of chromatin compaction did not show a significant difference between KO and WT (Fig. 1G). Additionally, sperm motility parameters determined by CASA were comparable between WT and KO sperm (Supplementary Fig. S4C and D). In order to further understand the underlying cause behind the infertility of KO mice, we tested the ability of *Actl7a^−/−^* sperm to undergo capacitation. KO and WT sperm were subjected to a 90-min incubation in BSA-rich HTF media to promote capacitation, then assessed by immunofluorescent localization of tyrosine phosphorylation which has been reported to localize to the sperm flagellar midpiece in capacitated sperm (Visconti *et al.*, 1995; Si and Okuno, 1999; Ded *et al.*, 2013). We observed that both WT and *Actl7a* KO sperm showed a normal tyrosine phosphorylation pattern in the head region of non-capacitated sperm, and a normal propensity to induce tyrosine phosphorylation on the midpiece of the sperm’s flagellum, indicative of proper capacitation after a 90-min incubation in BSA enriched HTF medium (Fig. 1J).

### *Actl7a* KO mice lack acroplaxome-associated F-actin and exhibit defects in proper acrosomal adherence to the nucleus

**Figure 2.**
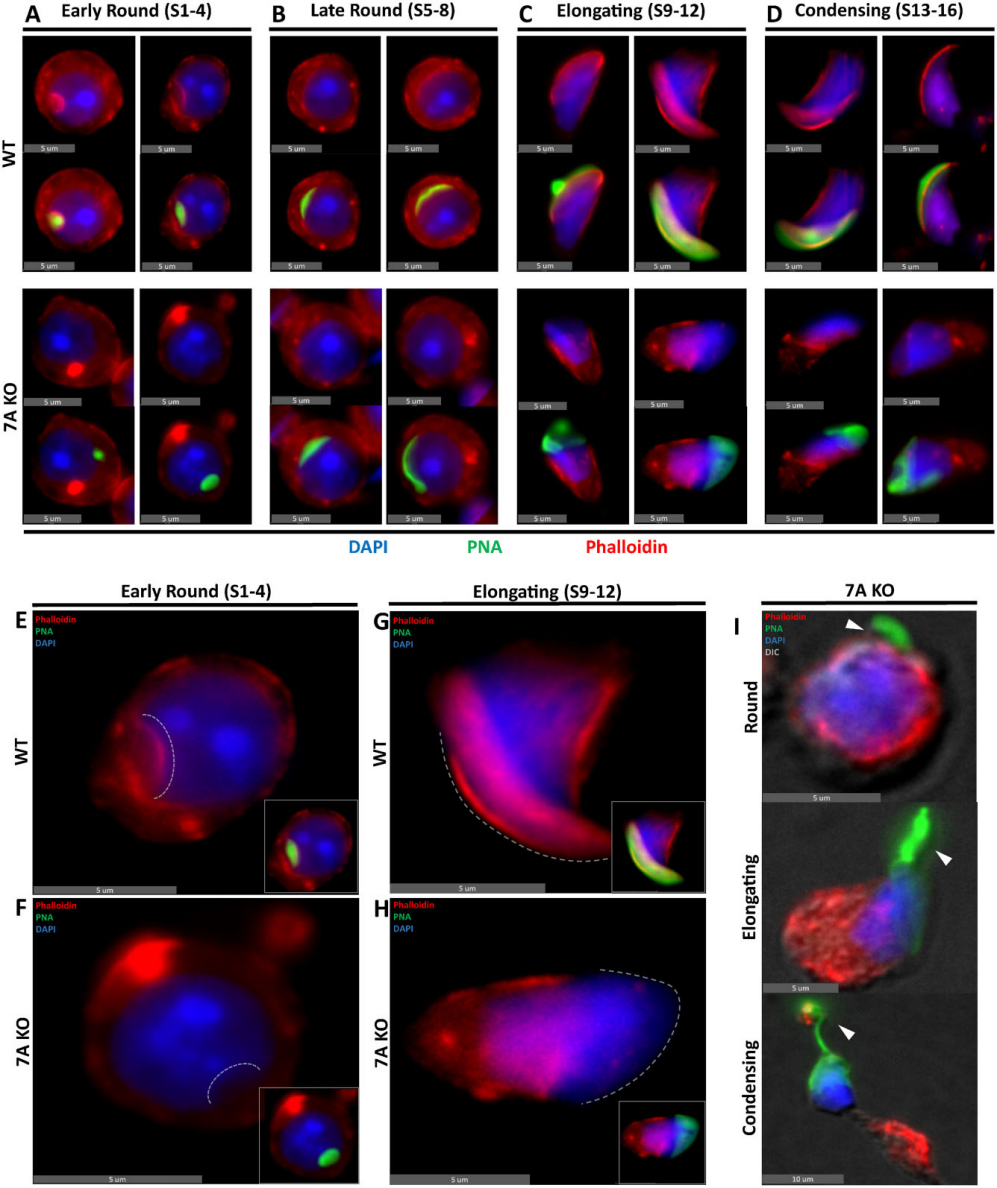
**Acrosomal instability is associated with loss of F-actin in the acroplaxome of *Actl7a* KO mice.** (**A**–**D**) *Actl7a* KO and WT singularized spermatids showing a lack of F-actin staining specifically in the acrosomal region of KO spermatids shown by the lack of phalloidin below the peanut agglutinin (PNA) stain. (**E**–**H**) Enlarged panel figure from subpanel A and C showing a complete lack of subacrosomal F-actin. Acrosomal attachment area is delineated by a dashed white line. (**I**) KO spermatids at different developmental steps showing a representative progression of acrosomal detachment as indicated by white arrowheads. ACTL7A, actin-like 7A; KO, knock-out; WT, wild type.

Next, we looked at the formation of the acrosome and underlying associated acroplaxome during spermiogenesis utilizing singularized spermatids. Using PNA as an acrosomal marker, and the F-actin stain phalloidin as a general cytoskeletal indicator, we observed that phalloidin stains the acroplaxome in WT spermatids. However, there is a complete loss of phalloidin-stained F-actin structures in the subacrosomal region of all observed *Actl7a* KO spermatids across all developmental steps (Fig. 2A–H). This signifies that ACTL7A is required for proper subacrosomal F-actin formation and/or stability as observed in WT spermatids. Interestingly, F-actins are still observed in other intracellular locations in the spermatids, indicating that the ACTL7A dependence of observable F-actin is limited to specific subcellular structures. Additionally, despite the clear lack of F-actin structures below the acrosome of KO spermatids, an acroplaxome-associated nuclear indentation is still observed in early capping phase round spermatids (Fig. 2E and F). These results indicate that neither ACTL7A nor F-actin is a prerequisite for the nuclear recession associated with normal acrosomal vesicle implantation and expansion and suggest that there are at least two different structural layers to the acroplaxome, one which is comprised of F-actin structures and another one which is neither made of F-actins nor dependent on their presence.

**Figure 3.**
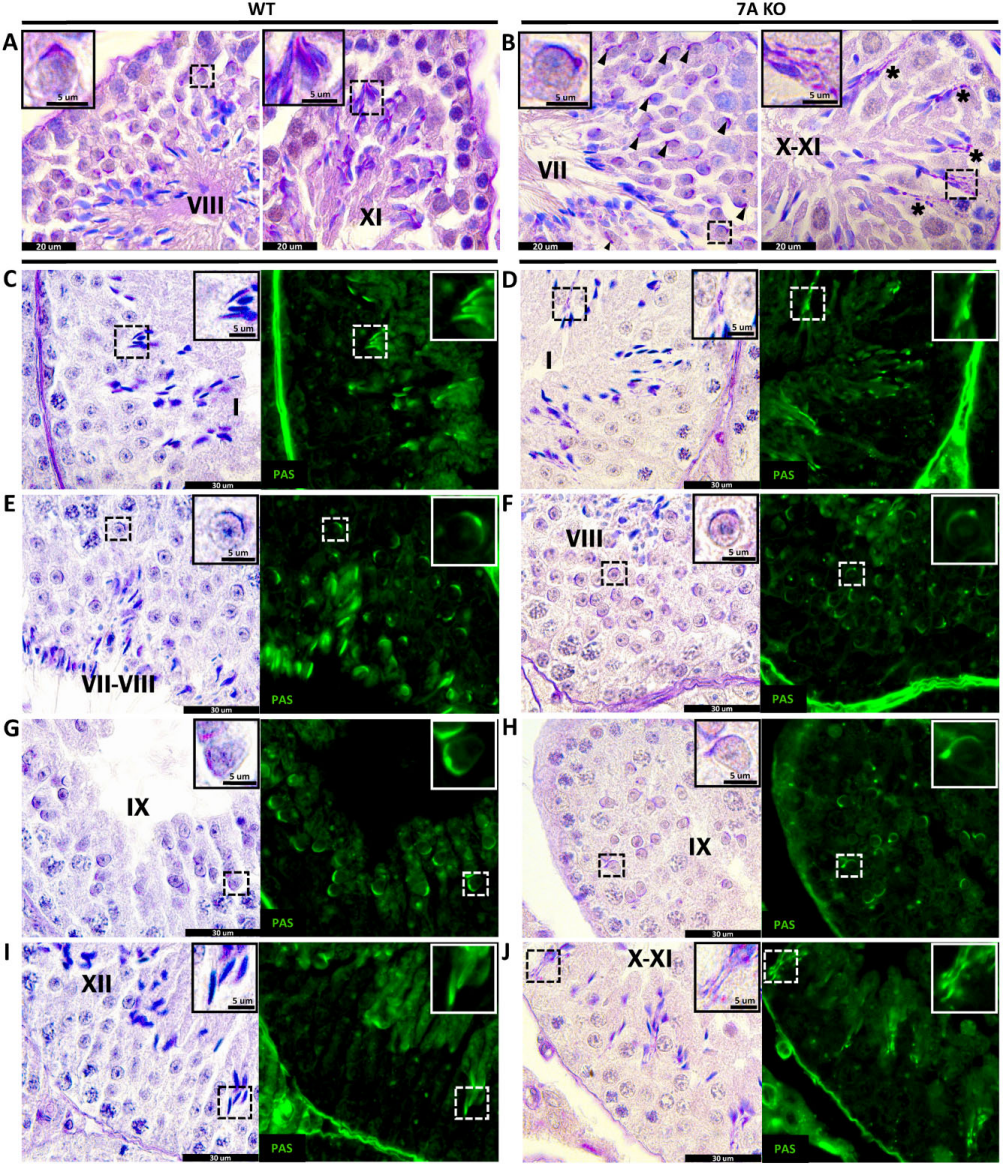
**Acrosomal defects found within the testis histomorphology of *Actl7A* KO mice.** (**A** and **B**) Periodic acid Schiff’s and hematoxylin (PAS-H)-stained PFA fixed testis cross-sections of KO and WT testis showing acrosomal granule displacement (arrowheads, B) and peeled acrosomes in Sertoli cell crypts (asterisks, B) as a characteristic phenotype of *Actl7a* KO mice. (**C**–**J**) PAS-H-stained Bouin’s fixed testis cross-section showing both chromogenic and fluorescent (shown in green) PAS signal, depicting displaced acrosomal granules and peeled acrosomes in the morphologically preserved seminiferous epithelium of *Actl7a* KO mice across multiple spermatogenic stages of development. ACTL7A, actin-like 7A; KO, knock-out; WT, wild type.

Complementing the loss of subacrosomal F-actins, we observed structural acrosomal instability starting in early round spermatids of the KO which further propagated at later stages of spermiogenesis (Fig. 2I). Taking a closer look at gross acrosomal defects in WT and KO singularized spermatids across three mice per genotype and over 200 cells analyzed per mouse revealed a clear increase in the incidence of acrosomal defects in the KO over the WT control. In early round spermatids (Steps 1–4), morphological defects of the acrosome, including its initial separation from the nucleus and fragmented acrosomal vesicles (Supplementary Fig. S5A–C), was observed in 23.68% of cells compared to 11.57% of cells in the WT; while late round spermatids (Steps 5–8) observed a bigger difference in notable acrosomal defects (Supplementary Fig. S5D and G–H) at a rate of 58.68% of KO cells compared to 12.43% in the WT. More drastic acrosomal abnormalities were observed in elongating and condensing spermatids with these cells exhibiting acrosomes peeling off or appearing to have been pulled by an external force as seen in individualized spermatids (Fig. 2I and Supplementary Fig. S5J). In addition, acrosomes of elongating and condensing spermatids consistently appear to be anomalous in the distribution of their content having a splotched PNA staining complexion with weakly stained areas where the nuclear DAPI is no longer covered by the PNA acrosomal stain (Fig. 2C and H and Supplementary Fig. S5E, F, and I). Finally, at later stages, condensing spermatids can be found with large portions of their acrosome missing from the sperm’s head (Fig. 2D). Overall, while acrosomal shaping defects caused by abnormal nuclear morphology of missing perforatorium were observed in 100% of KO sperm, other acrosomal defects were also observed at an increased rate of 75.44% of the time in KO elongating spermatids (Steps 9–12) compared to 10.91% in WT, and 84.85% in KO condensing spermatids (Steps 13–16) compared to 11.11% in WT. To ascertain whether the observed acrosomal abnormalities in individualized spermatids were exacerbated by the methodology used to separate the already compromised KO spermatids from the seminiferous epithelium, we stained Bouin’s and PFA fixed *Actl7a* KO paraffin-embedded testis sections with periodic acid Schiff’s reagent and hematoxylin (PAS-H) to see if we could detect similar acrosomal defects in whole fixed testis (Fig. 3). PAS-H staining on Stage VII–IX seminiferous tubules revealed late round and early elongating spermatids to have a consistent asymmetrical migration of the acrosomal granule to the edge of the acrosome in KO (Fig. 3E–H) compared to normal symmetrical localization in WT (Fig. 3A and B), comparable to the phalloidin-stained singularized late round spermatids (Fig. 2B). In WT round spermatids, the acrosomal granule is described as a dense glycoprotein-rich globular body housed within the acrosomal vesicular matrix that is always anchored to the inner acrosomal membrane at the center of the acrosome (Russell *et al.*, 1993). Seeing its consistent displacement in *Actl7a* KO spermatids is most unusual, and suggests that, in part, the anchoring mechanism for the acrosomal granule has gone awry. Surprisingly, we also observed what appeared to be spermatid-dissociated PAS positive acrosomal fragments congregating in Sertoli cell crypts in stage X-I seminiferous tubules (Fig. 3B, D, and J). A closer look at individual spermatids in Bouin’s fixed testis section (Fig. 3C–J) revealed a clearer progression of the acrosomal pathology associated with torn acrosomes and displaced acrosomal granules observed in ACTL7A deficient mice. Across three mice per genotype and over 200 quantified cells per mouse, we observed that early round spermatids (Steps 1–4) were found to have peeling acrosomes and/or displaced acrosomal granules 13.66% of the time in the KO compared to 7.89% of the time in WT; 60.13% in late round spermatids (Steps 5–8) compared to 8.43% in WT; 61.11% in elongating spermatids (Steps 9–12) compared to 8.59% in WT; and 80.42% in condensing spermatids (Steps 13–16) compared to 7.41% in WT. The observed increasing incidence of acrosomal defects, in both enzymatically singularized and testicular spermatids, positively correlates with the morphological development of the acrosome which is suggestive of two possibilities: (i) that ACTL7A might not play an important role on the coalescence and adherence of proacrosomal vesicles to the nucleus during the Golgi phase of development, but rather that ACTL7A is a stabilizing factor during acrosomal capping and later stages of acrosomal morphogenesis; or (ii) that ACTL7A is required throughout the entirety of acrosomal development but its absence forcefully compounds more apparent acrosomal defects as spermatids develop without ACTL7A. Overall, the phenotypic similarity found in PAS-H-stained testis sections, singularized spermatids, and epididymal sperm congruently implicate the importance of ACTL7A in acrosome granule adherence and acrosomal stability, thus delineating a likely explanation for the cause of *Actl7a* KO mouse infertility.

### ACTL7A localization to different intracellular compartments is stage-dependent and varied across spermatogenesis

**Figure 4.**
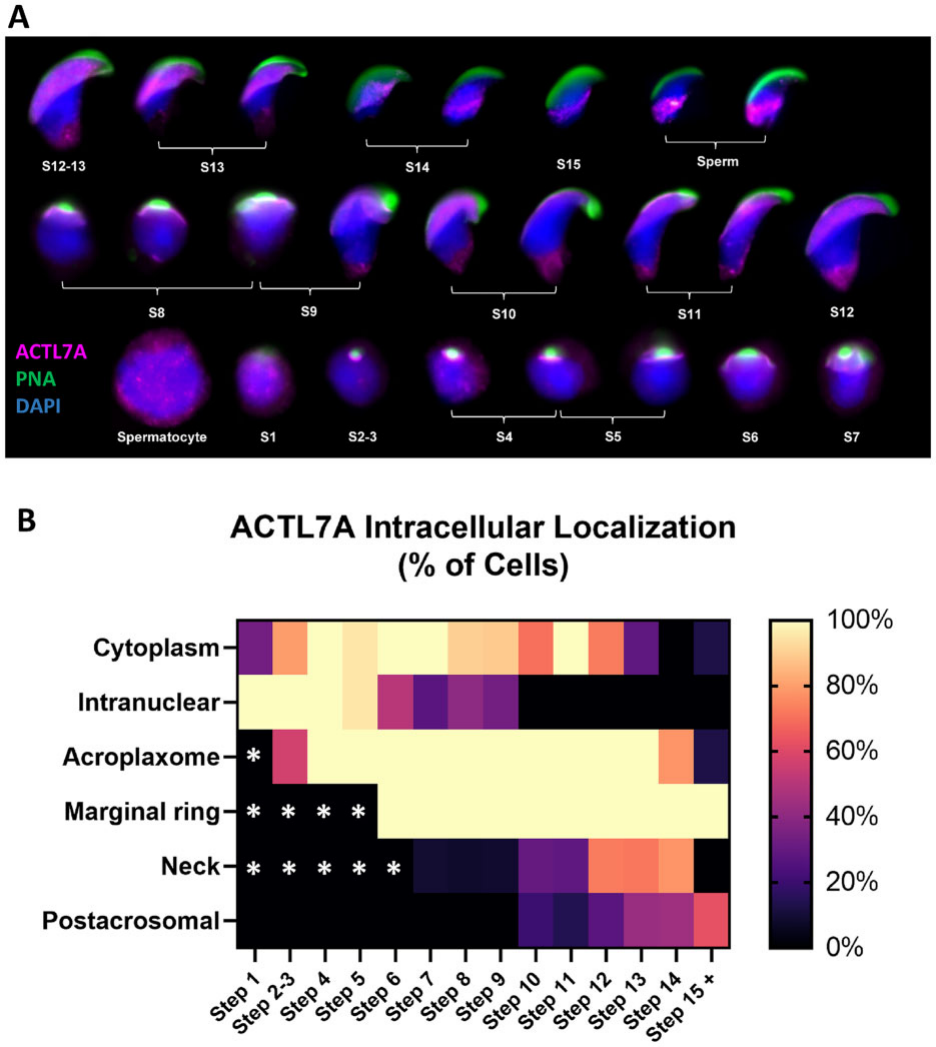
**Dynamic localization of ACTL7A across spermatogenesis.** (**A**) Paneled figure showing spatiotemporal expression and localization of ACTL7A across spermatogenic development, spermatids Steps 1–15 (S1–15), through widefield fluorescent microscopy. (**B**) Heatmap demonstrating the percentage of cells of a particular spermiogenic step having ACTL7A localized to different cellular regions from three separate mice. White asterisks denote cellular structures which have not yet been formed for that developmental step. ACTL7A, actin-like 7A.

**Figure 5.**
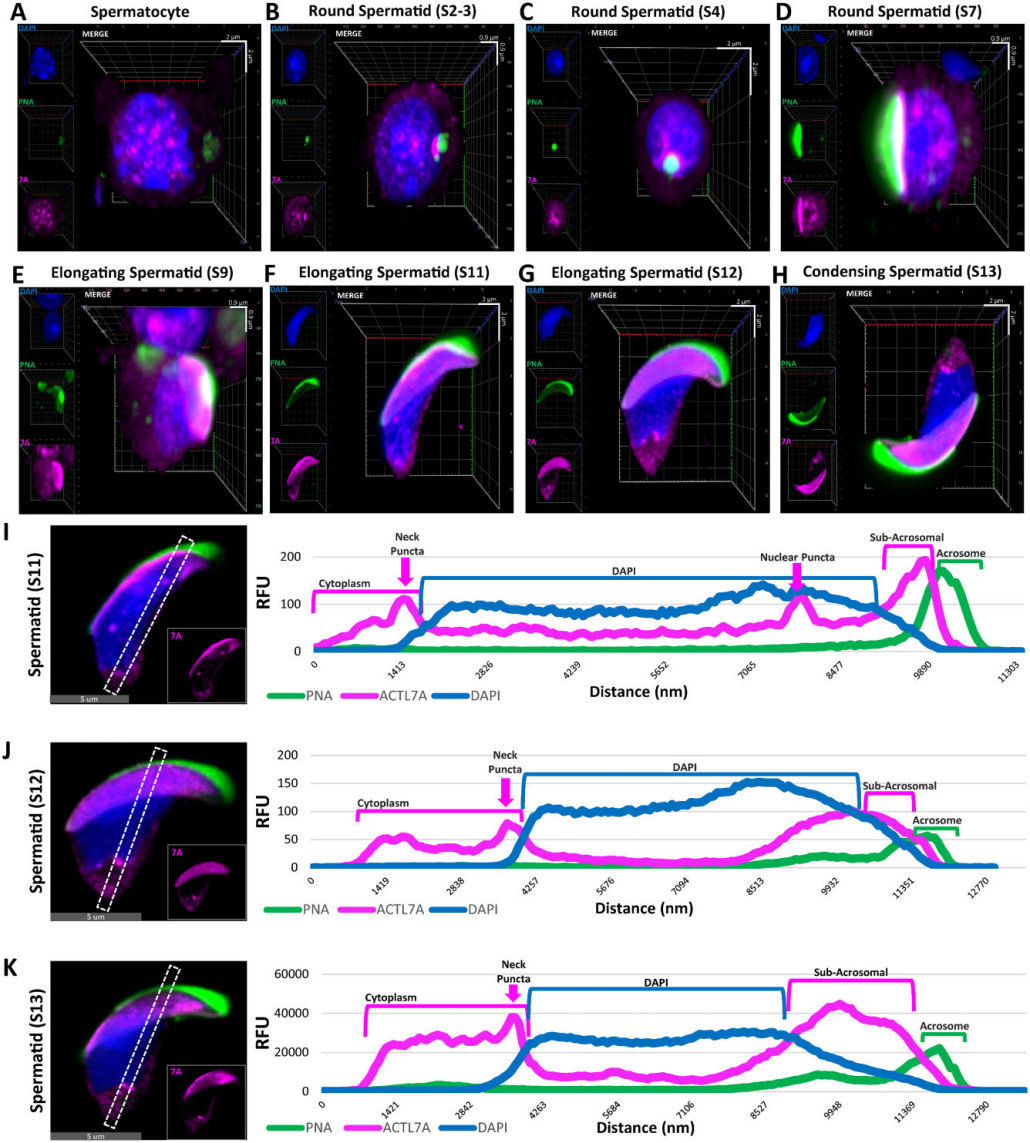
**Intranuclear presence of ACTL7A.** (**A**–**H**) Three-dimensional (3D) rendered confocal z-stacks of spermatids at different developmental steps illustrating a clear intranuclear localization pattern of the protein in spermatocytes, round spermatids, elongating spermatids, and condensing spermatids. Scale bar of the embedded 3D grid is enhanced with X and Y scale bars. (**I**–**K**) Representative line scan analysis of elongating spermatids from subpanels F–H across their most intranuclear optical cross-section, depicting ACTL7A positive puncta in the neck region of Steps 11–13 spermatids and intranuclear puncta in Step 11 spermatids demarcated by the graphed changes in relative fluorescent units (RFUs) as a measurement of fluorescent intensity along the length of the line scan. ACTL7A, actin-like 7A.

Following our observations of the *Actl7a* KO phenotype, we wanted to further investigate the intracellular localization of ACTL7A throughout spermatogenesis to better understand the potential roles it might be involved in, based on where it is located within the germline. ACTL7A localization on testis sections (Supplementary Fig. S6) is germ-line specific, consistent with expression profiles in publicly available databases such as the Human Protein Atlas Database single-cell sequencing datasets (Karlsson *et al.*, 2021). Individualized germ cell preps of WT murine testis were obtained by enzymatic dispersion and subjected to immunofluorescent microscopy. The various intracellular localizations of ACTL7A were noted (Figs 4 and 5) and the percentage of cells exhibiting each intracellular localization pattern for each step of spermatid development was quantified (Fig. 4). We initially noted ACTL7A to have five major patterns of intracellular localization: being nuclear associated from spermatocytes to Step 9 spermatids, in the acroplaxome from Steps 2 to 13 spermatids, present in the marginal ring from Steps 6 to 15+ spermatids, having flagellar connecting piece regional localization from Steps 7 to 14 spermatids, and being postacrosomal from Step 10 spermatids to spermatozoa (Fig. 4). Moreover, the expression patterns of ACTL7A appear to surge and wane in their observed frequency within different cellular regions dependent on the spermatogenic step (Fig. 4B). Cytoplasmic expression of ACTL7A was observed in a fraction of the spermatocyte and early round spermatid populations, with cytoplasmic expression frequency increasing to be in nearly all late round spermatids. The prevalence of cytoplasmic localization again decreases as nuclear elongation and condensation occur (Fig. 4B). In contrast, nuclear association of ACTL7A is found in spermatocytes and early round spermatids and incidence of its nuclear association gradually decreases to a complete absence as spermatid elongation progresses (Fig. 4B). A similar pattern of gradual change was also observed with the prevalence of postacrosomal and neck region-associated ACTL7A, except that these patterns only begin to be detected during elongation and further intensify during spermatid condensation (Fig. 4B).

Step-specific intracellular localization patterns of ACTL7A were confirmed by the same subset of indirectly labeled fluorescent WT spermatids for confocal analyses of spatial signal intensity. Optical cross-section z-stacks of representative spermatids from distinct spermatogenic steps were rendered as three-dimensional models to better visualize their cellular structure (Fig. 5A–H). In analyzing spermatocytes as well as round spermatids, we discerned that the aforementioned nuclear association of ACTL7A was indeed mostly intranuclear rather than peri-nuclear, having a blotched nuclear pattern. Additionally, we performed line scans on single optical cross-sections through a central plane of the nucleus (that which delineated the entire shape of the nucleus and was 1–2.5 µm deep from the nuclear equatorial surface) of Steps 11, 12, and 13 spermatids (Fig. 5I–K). Longitudinal trans-nuclear line scan analysis shows the relative fluorescence units (RFUs) for each fluorophore localized in these spermatids and confirmed the localization of ACTL7A to the subacrosomal and the neck regions but also showed a distinct change in the intranuclear presence of ACTL7A in elongating spermatids indicated by changes in RFUs (Fig. 5I–K). While intranuclear ACTL7A in spermatocytes and round spermatids adopted an amorphous blotched pattern, elongating spermatids had a singularized puncta pattern through Step 11 after which ACTL7A was not found in the nucleoplasm of more mature spermatids (Fig. 5I–K).

### ACTL7A perturbs nuclear morphology and recapitulates germ cell-like structural and spatial localization patterns when overexpressed in HEK293F cells

**Figure 6.**
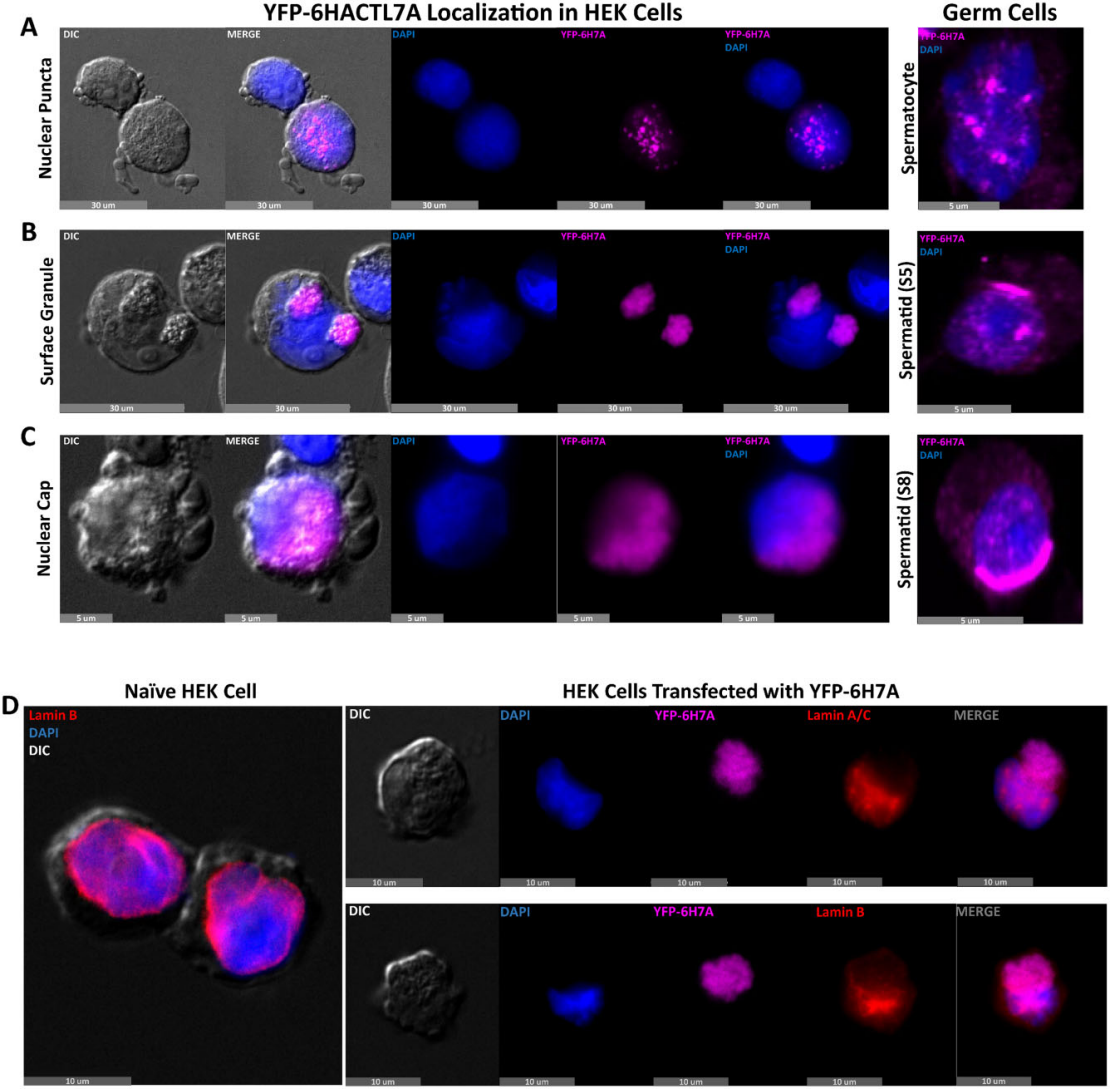
**Varied nuclear intrusions of ACTL7A in transfected HEK293F cells.** HEK293F cells transfected with a YFP-tagged ACTL7A recombinant plasmid showing three distinct localization patterns of the fusion protein: having a nuclear puncta pattern mostly resembling that of spermatocyte localization (**A**), a surface granule pattern akin to the ACTL7A containing acroplaxome of round spermatids (**B**), and a nuclear cap pattern reminiscent of late phase round spermatids (**C**). (**D**) Nuclear Lamin stains of both naïve and transfected HEK293F cells showing a likely displacement of the nuclear envelope by ACTL7A surface granules. ACTL7A, actin-like 7A.

To determine whether ACTL7A retained some of its natural propensity to localize to specific intracellular compartments in an exogenous system, we generated a YFP-6His-ACTL7A (Y6H7A) fusion protein vector in a pcDNA6.2 plasmid backbone and transfected HEK293F cells to observe the expression and localization of the overexpressed protein (Supplementary Fig. S1A). We found that overexpression of ACTL7A was lethal to the HEK293F cells which exhibited a 66% viability after 24 h (Supplementary Fig. S1C), compared to >98% viability in cells transfected with a YFP-only control vector after the same time period of incubation. We therefore assessed morphology at 14 h at which time cell viability was >80% in Y6H7A transfected cells and >98% in control transfected cells. Assessment by widefield fluorescent microscopy on these Y6H7A transfected HEK293F cells revealed that ACTL7A had three distinct localization patterns reminiscent of those found in spermatocyte and round spermatid stages of germline development (Fig. 6A). Across three individually transfected cultures, the blotched nuclear pattern found in spermatocytes was detected in 7% of analyzed HEK cells, while 61% of transfected cells had a surface granule vicinal to the nucleus, or a nuclear cap pattern evocative of round spermatids in the remaining 32% of transfected HEK cell respectively (of ∼100 cells scored from three biological replicates) (Fig. 6A–C). We also note that when ACTL7A localized in a surface granule or nuclear cap pattern, a granule was also observed in the HEK293F cell by DIC microscopy (Fig. 6A–C). Furthermore, indirect fluorescent labeling of nuclear Lamins in transfected HEK cells strongly suggests the surface granule pattern of ACTL7A to be associating with and perturbing the nuclear envelope, much like how the acroplaxome-associated nuclear envelope becomes concave in Steps 2–4 spermatids (Fig. 6D). These observations revealed that ACTL7A is sufficient to actively remodel nuclear shape from its exterior in transfected HEK293F cells but is not entirely required in spermatids, is not dependent on other testis-specific factors for intranuclear transport and is capable of self-aggregation within mammalian cells.

### ACTL7A interacts with varied cytoskeletal proteins

**Figure 7.**
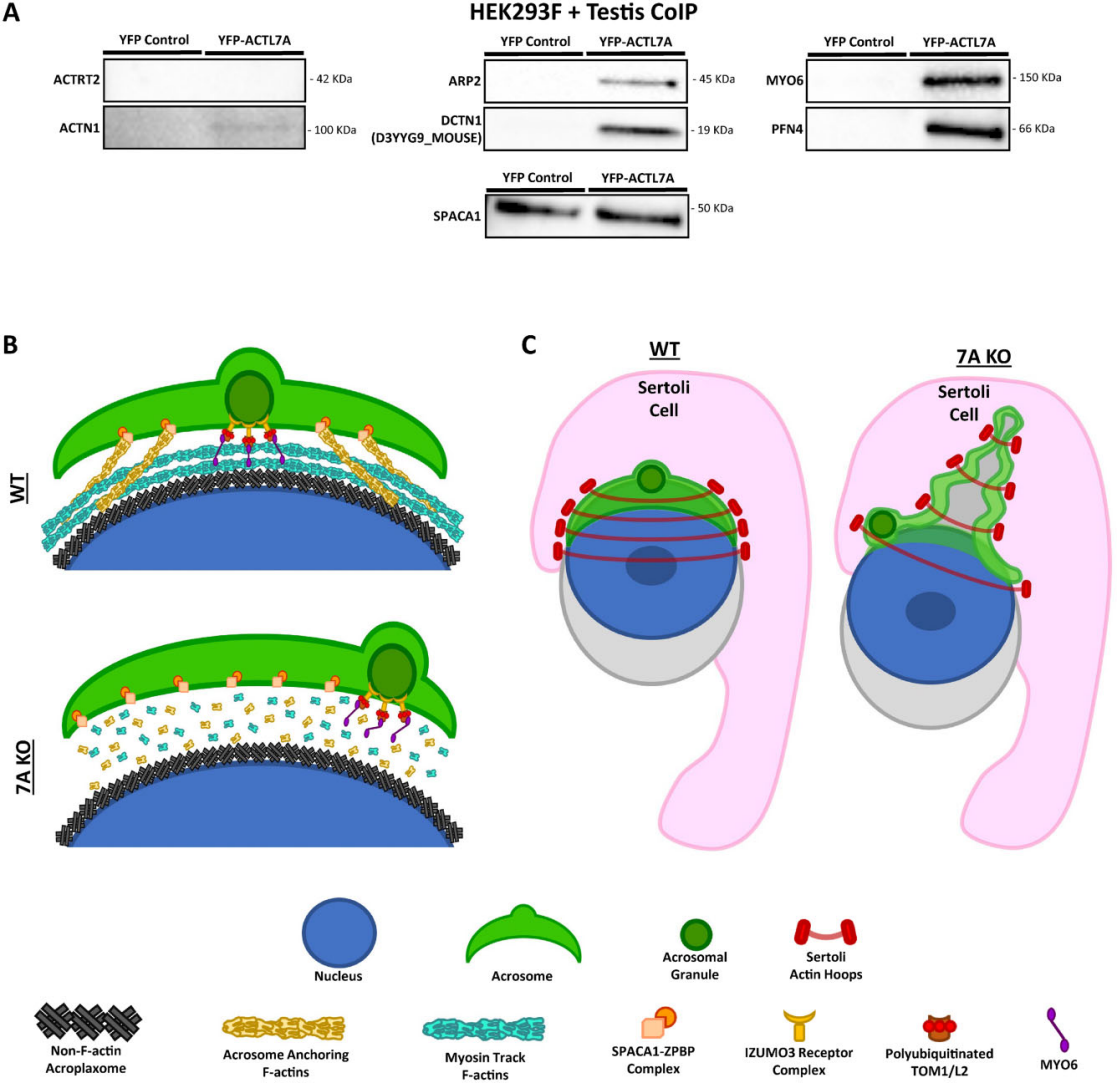
**Putative binding partners of ACTL7A and phenotype model.** (**A**) Coimmunoprecipitation (Co-IP) products of HEK293 expressed YFP-ACTL7A cell lysate incubated with adult testis lysate. Co-IP YFP-ACTL7A with anti-GFP camelid nanobody coated agarose beads. (**B**) Intracellular model of the subacrosomal space depicting the attachment of the granule-anchoring MYO6 motor protein complex to ACTL7A-dependent F-actins and their organizational disruption in the absence of ACTL7A. (**C**) Cellular diagram portraying a model as to why acrosomal fragments detached from spermatids and retained in Sertoli cell crypts in *Actl7a* KO mice. ACTL7A, actin-like 7A; KO, knock-out.

To better understand the potential roles of ACTL7A in acroplaxome stability and biogenesis, we performed coimmunoprecipitations with YFP-tagged ACTL7A produced by overexpression in transfected HEK293F cells. Using the YFP tag on our fusion protein product to facilitate coimmunoprecipitation (Co-IP), we screened for potential interacting partners of ACTL7A by incubating the soluble fraction of transfected HEK293F cells and detunicated testis soluble fraction lysates. Western blot analysis of the Co-IP products did not identify interactions between ACTL7A and ACTRT2, and only minimal potential interaction with α-actinin (ACTN1) (Fig. 7A and Supplementary Fig. S7). In contrast, by using ACTL7A as a molecular bait, we were able to identify its interaction with ARP2, a 19-kDa band pertaining to dynactin p150 glued (DCTN1), MYO6, and PFN4 (Fig. 7A and Supplementary Fig. S7). These interactions are specific to Co-IP with testis lysate and were not detected when YFP-7A Co-IP was performed directly from HEK cells (Supplementary Fig. S8). This indicates that either HEK cells do not express these proteins, or the interactions require additional testis-specific factors. We also performed a densitometric analysis of total protein products in Co-IP from YFP-only expressed protein with testis lysate to demonstrate non-ACTL7A background interactions, and YFP-ACTL7A Co-IP directly from HEK cells, which would indicate proteins expressed in HEK cells that can associate with ACTL7A in the absence of additional testis-specific proteins, and from the YFP-ACTL7A plus testis lysate Co-IPs (Supplementary Fig. S8). Densitometric assessment of the Co-IP products demonstrated a number of bands specific to the experimental Co-IP of interest. We also performed western blots to assess interactions between SPACA1 and ACTL7A as previously reported (Chen *et al.*, 2021); however, we were unable to detect specific interactions given that a specific band for SPACA1 was observed in both our YFP-negative controls in our experimental conditions, thereby suggesting that there may be an insoluble precipitating complex containing SPACA1 or a nonspecific association confounding the assay and precluding detection of specific interactions as previously reported (Chen *et al.*, 2021) (Fig. 7A and Supplementary Fig. S7). Considering the varying cytoskeletal interacting partners identified for ACTL7A and its essential role in acrosomal adherence and subacrosomal F-actin association, we propose that the top-most layer of the acroplaxome is enriched with ACTL7A-dependent F-actins tracks, on which the previously described acrosomal granule adherent MYO6 motor protein complexes (Zakrzewski *et al.*, 2020) are attached (Fig. 7B and Supplementary Fig. S7). In addition, given the conserved phenotype of peeling acrosomes found in *Actl7a* KO mice, we suggest that the anchoring complexes which maintain acrosome associations with the acroplaxome are also ACTL7A dependent and are unable to properly form in KO spermatids. We propose that the lack of these stabilizing connections subsequently allows the apical ectoplasmic specialization and constricting actin hoops of the Sertoli cells to pinch off and pull the compromised acrosome during the initiation of spermatid elongation (Fig. 7B and C and Supplementary Fig. S7), which would then explain their observed detached localization within the Sertoli cell crypts.

## Discussion

A conspicuous observation and significant finding of this research are that in the subacrosomal space of *Actl7a* KO mouse spermatids, there is a lack of F-actin. The KO spermatids did not lose F-actin in other cellular regions, indicating that ACTL7A is not globally required for F-actin formation within developing spermatids and emphasizing that ACTL7A is a hyperspecialized ARP, required for F-actin formation, regulation, and/or stability in the subacrosomal space. To the best of our knowledge, this is the first time a tissue-specific ARP family member has been shown to be essential for the presence of F-actin at a specific subcellular space in only a single specialized mammalian cell. This suggests a mechanism that may explain previously reported negative effectors of infertility associated with acrosomal dissociation downstream of gene mutations in mice and humans alike and points to a novel role for the ARP family of proteins.

Somatic ARPs are generally expressed more broadly and affect cytoskeletal or nucleoskeletal dynamics in a variety of contexts. Somatic cell expressed members of the ARP family are known to form filaments and contribute to F-actin nucleation and/or to F-actin stability. Filament-forming actins include conventional alpha, beta, and gamma actin (Gunning *et al.*, 2015). Similarly, ARP1 forms a short eight-subunit filament within the dynactin protein complex that also includes monomers of β-actin and ARP11 (Urnavicius *et al.*, 2015). Other somatic ARPs do not form filaments themselves but can instead influence F-actin dynamics. For example, ARP2/3 complexes help to nucleate actin filaments and can attach to actin filaments to serve as branch points in branched actin mesh networks (Goley and Welch, 2006). Apart from intrinsic F-actin formation, all other known roles of ARPs involve associations in large multimeric protein complexes, suggesting that ACTL7A would operate in a similar fashion. As such, it is not yet clear whether ACTL7A may be the filament-forming actin in the acroplaxome, or whether its role is to nucleate, stabilize formation, and/or anchor F-actin formed by conventional ARPs to the nuclear surface. To begin to understand the associations of ACTL7A with ubiquitous nuclear envelope proteins that might be required for acrosomal anchoring, we overexpressed ACTL7A in the immortalized mammalian embryonic kidney HEK293F cell line. HEK293F cells lack an acroplaxome and other spermatid-specific features with which ACTL7A associates; nonetheless, we observed that ACTL7A localized to nuclear and perinuclear regions, as intranuclear blotches, and as surface aggregates similar to its observed spermatid localization patterns. This suggests that ACTL7A localization and attachment in these cellular regions do not require other sperm-specific proteins or structures, and that ACTL7A is sufficient to induce nuclear reorganization in a non-germline cell culture. It is not yet clear if this required somatic chaperones and/or other translocation mechanisms conserved across mammalian cells, or more specifically, conserved between germline and HEK293 cells where these localization patterns were observed. Given our observations, we propose that ACTL7A forms a unique testis-specific protein complex to anchor and stabilize acrosomal adherence to the acroplaxome at least in part by facilitating the presence of F-actin in the subacrosomal space.

In order to further elucidate the roles ACTL7A plays in spermatid cytoskeletal regulation, we investigated its molecular associations and interactions with other cytoskeletal proteins both present within and outside the subacrosomal space via coimmunoprecipitation. Surprisingly, ACTL7A interaction with ACTRT2 was not detectable by Co-IP (Fig. 7A and Supplementary Fig. S7) despite ACTRT2 being present in the acroplaxome and implicated to be a structural component (Zhang *et al.*, 2022a,b). This observation could be due to an inability to coprecipitate these proteins in the soluble fraction but may also indicate a necessity to interact in a larger complex of acroplaxome protein components. The latter interpretation rather suggests that there is a heterogeneity of large protein complexes forming in the acroplaxome with unique components. Within the subacrosomal space, both the ARP2/3 complex and myosin motors are expected to bind directly to actin filaments for F-actin branching and motor transport respectively based on their known roles in somatic cells and their effects in KO models (Goley and Welch, 2006; Zakrzewski *et al.*, 2020). Therefore, the strong association of ACTL7A with both ARP2 and MYO6 (Fig. 7A and Supplementary Fig. S7) is suggestive that either ACTL7A forms the subacrosomal F-actin structures or is a monomeric contributor to filament formation and/or stability. Coprecipitation of MYO6 with ACTL7A is further illuminating, as it provides an explanation for the observed displacement of the acrosomal granule in *Actl7a* KO spermatids (Figs 2B and 3B). Our reported acrosomal granule displacement phenotype strongly resembles that of previously described IZUMO3 (Inoue *et al.*, 2021) and MYO6 (Zakrzewski *et al.*, 2020) null mice. A clear distinction between the granular displacement of *Izumo3* and *Myo6* KO mice is that the acrosomal granule does associate with the inner acrosomal membrane in *Myo6* KO spermatids but is not symmetrically anchored to acroplaxome F-actins (Zakrzewski *et al.*, 2020), permitting the inner membrane-associated granule to move about the acrosomal vesicle. In contrast, *Izumo3* KO spermatids have a complete lack of granular adherence to the inner acrosomal membrane (Inoue *et al.*, 2021) and, as such, they remain afloat in the acrosomal vesicle. Taking into account these previous observations together with our own data, we propose that an IZUMO3-containing protein complex is responsible for acrosomal granule anchoring to the inner acrosomal membrane and it also links to the subacrosomal poly-ubiquitinated TOM1/L2-containing MYO6 motor (Zakrzewski *et al.*, 2020), which we propose is tethered to an ACTL7A-dependent F-actin acroplaxome network (Fig. 7B and Supplementary Fig. S7). In addition to the acrosomal granule displacement defects, we also observed acrosomes peeling off in *Actl7a* KO spermatids, suggesting that ACTL7A-dependent F-actins also play a structural role for acrosomal anchoring and development, as is also reflected in our proposed model showing the subacrosomal space devoid of F-actins in the absence of ACTL7A (Figs 2F and 7B). Also, the actin-binding and filament bundling protein ACTN1 was shown to have a weakly detectable affinity to ACTL7A (Fig. 7A and Supplementary Fig. S7) through Co-IP. This weak interaction could implicate ACTL7A to be an indirect effector of sperm fertilization competence, given that ACTN1 is found on both sides of the acrosomal membrane and is known to interact with other focal adhesion proteins which regulate acrosome reaction (Roa-Espitia *et al.*, 2016), which is imperative for the sperm’s ability to penetrate the zona pellucida for fertilization.

In addition to the role ACTL7A plays in acroplaxome formation, acrosome attachment, and acrosome granule stability, our ACTL7A intracellular localization data indicate that this protein may have additional functions in spermatid maturation and sperm. Our observation that ACTL7A is present in the intranuclear compartment, acroplaxome, marginal ring, neck region, and later the postacrosomal region (Fig. 4A) might suggest roles in these other locations as well as a sense of directionality with regard to its migration between these regions. However, we did not observe defects in flagellar integrity (Fig. 1C and J), in the ability to capacitate (Fig. 1J), or in sperm motility (Supplementary Fig. S4), which suggests that these cellular processes are independent of ACTL7A function. Interestingly, we did see an increase in TUNEL positive sperm despite not seeing differences in sperm motility nor chromatin accessibility by CMA3 assay (Fig. 1F–G) nor defects in sperm head compaction (Fig. 1C). Although TUNEL staining is classically used to indicate cell death, it is actually a measure of DNA damage. Given the known role of somatic ARPs in chromatin remodeling, it is possible that a loss of ACTL7A leads to loss of chromatin integrity which leads to DNA damage without a loss of viability or motility. Given the normal sperm counts and the CMA3 assays indicating normal compaction of *Actl7a* KO sperm chromatin, the data taken together are suggestive of a role for ACTL7A in mediating chromatin integrity during spermiogenesis after checkpoints that may reduce spermatogenic capacity, but before DNA compaction is complete. However, it is reasonable that ACTL7A has specific functions in these other cellular compartments (some of which are beginning to be brought to light with reported observations of phospholipase C zeta (PLCζ) not localizing properly to the postacrosomal sheath in dysfunctional ACTL7A knock-in mouse sperm (Dai *et al.*, 2022)); yet, the mechanism of action behind ACTL7A function in, and translocation to, these regions is currently unknown. As to what might be driving ACTL7A migration across different cellular regions, we speculate based on our step-specific localization (Fig. 4A) and Co-IP data (Fig. 7A and Supplementary Fig. S7). If ACTL7A follows similar regulatory/functional rules to other ARPs, then one can envision monomerized ACTL7A to be shuttled from inside the nucleus to a cytoplasmic space via a similar mechanism by forming a trimeric complex with profilin and exportin (Kristó *et al.*, 2016), which could explain the abnormally large kDa PFN4-specific band that we observed to coprecipitate with ACTL7A (Fig. 7A and Supplementary Fig. S7). This interaction between PFN4 and ACTL7A is consistent with the previously reported localization of testis-specific PFN3 and PFN4 in rat spermatids, as it is shown to go from the subacrosomal space to the postacrosomal/manchette region after the initiation of nuclear elongation (Behnen *et al.*, 2009). Similarly, the coprecipitation of DCTN1 with ACTL7A (Fig. 7A and Supplementary Fig. S7) also suggests a possible mechanism of translocation to the postacrosomal region via the manchette given the abundance of dynein/dynactin motor protein complexes carrying protein cargo along microtubule tracks found in the area (Kierszenbaum and Tres, 2004; Kierszenbaum *et al.*, 2011). It is worth noting that the DCTN1 band observed in this study does not correlate in size with canonical DCTN1, but rather it matches a 19-kDa mouse-specific isoform D3YYG9 which is currently uncharacterized in testis.

More interestingly, we found that despite mature WT spermatozoa having an acroplaxome, ACTL7A is found almost exclusively on the postacrosomal sheath region of the sperm head in mice (Fig. 4A) and was previously reported to also be partially retained in the acroplaxome in humans (Chen *et al.*, 2021; Xin *et al.*, 2022). The partial retention of ACTL7A in the human acroplaxome might suggest a divergent function of the protein unique to primates, that the human postacrosomal sheath formation or function is not as reliant in ACTL7A activity, or simply that differences in methodologies and antibodies used may have yielded deviating results. Of note, we have consistently detected a single ACTL7A positive band via western blot from testicular lysates while a second lower kDa band appears exclusively in caudal sperm lysate (Fig. 1B), which can signify that cleavage of ACTL7A or removal of post-translational modifications are required to allow ACTL7A to adequately function in the postacrosomal sheath. It is known that during epididymal transit, sperm acquire motility and fertilization competence, signifying changes in which epididymosome-mediated modifications of sperm proteins, including protein cleavage, have been considered to be a contributing factor (Zhou *et al.*, 2018, 2019; Paul *et al.*, 2021) and could potentially explain the observed size discrepancy and potential proacrosomal function of ACTL7A in sperm. Even though we did not observe changes in motility and sperm capacitation in *Actl7a* KO sperm, further investigation is needed to see whether this protein plays additional roles in sperm reorganization events such as oocyte fusion.

In summary, this study has shown that the consistent localization of ACTL7A to the acroplaxome throughout spermiogenesis and the observed acrosomal defects in *Actl7a* KO mice strongly support that this testis-specific actin-like protein is a crucial component of acroplaxome biogenesis and acrosomal granule anchoring. The similarities between round spermatids and ACTL7A-transfected HEK cells, with regard to its general localization, are suggestive that ACTL7A is capable of natively interacting with the nuclear envelope and aggregating on the nuclear surface, thus resulting in a polarized nucleus. Despite its nuclear adherent and self-assembling tendencies, it stands to reason that ACTL7A requires other protein components to form a successful cytoskeletal scaffold to strengthen the acrosome-acroplaxome adherence interface through formation or regulation of subacrosomal F-actin. However, we have demonstrated here that there is strong evidence that the acroplaxome is comprised of at least two separate structural layers, that with and that without F-actins, and we have shown the indispensability of ACTL7A for the presence of subacrosomal F-actin, and for male fertility.

## Supplementary Material

gaad005_Supplementary_Data

## Data Availability

The data underlying this article are available in the article and in its Supplementary material.
