# Supplementary material for: Testis-specific actin-like 7A (ACTL7A) is an indispensable protein for subacrosomal-associated F-actin formation, acrosomal anchoring, and male fertility
Source: Mol Hum Reprod. 2023 Feb 3;29(3):gaad005. doi: 10.1093/molehr/gaad005 (PMC9976968; doi:10.1093/molehr/gaad005)
Supplement: gaad005_Supplementary_Data [file gaad005_supplementary_data.pdf]

Testis-Specific Actin-Like 7A (ACTL7A) is an indispensable protein for subacrosomal associated F-actin formation, acrosomal anchoring, and male fertility.

P. Ferrer [1,2], S. Upadhyay [2], M. Ikawa [3], T.M. Clement [1,2]

[1]Interdisciplinary Faculty of Toxicology Program, Texas A&M University, College Station, TX, USA [2]Department of Veterinary Physiology and Pharmacology, School of Veterinary Medicine and Biomedical Sciences, Texas A&M University, College Station, TX, USA [3]Department of Experimental Genome Research, Research Institute for Microbial Diseases, Osaka University, Suita, Osaka 565-0871, Japan

Table of Contents

- **Supplementary Figure S1:**
  YFP-TEV-6His-ACTL7A Plasmid construct and HEK239F transfection.
- **Supplementary Figure S2:**
  Antibody validation on spermatids and ACTL7A intracellular localization confocal analysis.
- **Supplementary Figure S3:**
  Western blot products for Actl7A KO validation.
- **Supplementary Figure S4:**
  *Actl*7A KO sperm exhibits high levels of DNA damage and but normal motility.
- **Supplementary Figure S5:**
  Observed acrosomal defects in WT and ACTL7A KO singularized spermatids.
- **Supplementary Figure S6**:
  ACTL7A localization by IHC is exclusively present in spermatids on WT testis sections.
- **Supplementary Figure S7**:
  Co-IP western blot products.
- **Supplementary Figure S8:**
  Co-IP western blot Controls and Silver Stain.
- **Supplementary Table SI:**
  Antibodies and Primers.

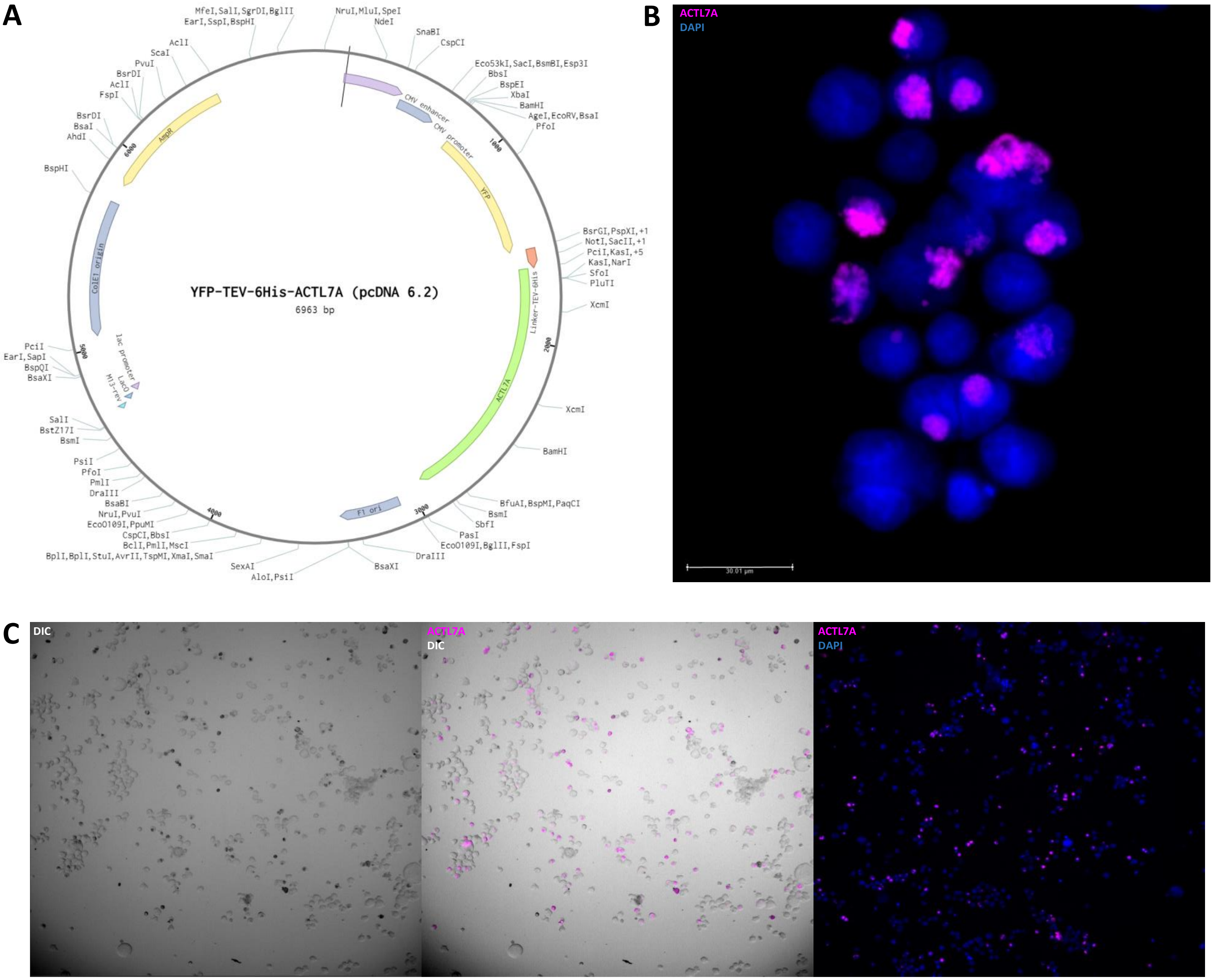

Culture Time: 24h **Transfection Vehicle:** Lipofectamine 293 **Viability:** 66% (n:182) **Efficiency:** 48% (n:423)

Supplementary Figure S1: YFP-TEV-6His-ACTL7A Plasmid construct and HEK239F transfection. (A) Diagram of the YFP-6His-ACTL7A plasmid construct used to transfect HEK293F cells. (B) HEK293F cells depicting varied ACTL7A localization patterns. (C) Widefield image depicting darkly contrasted spots on cells via DIC which are ACTL7A positive, showing the drastic morphological effect of the expressed protein in HEK293F cells.

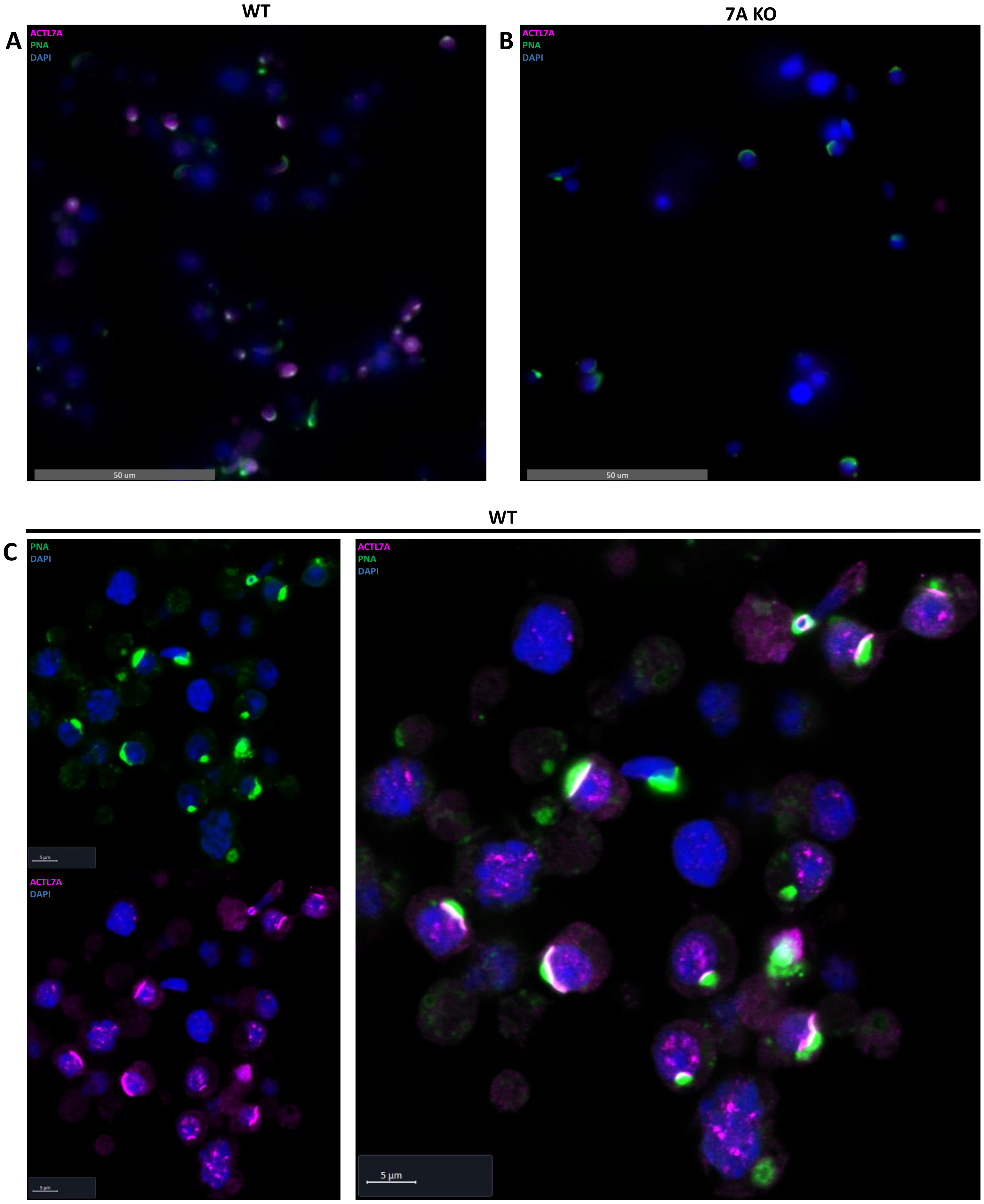

Supplementary Figure S2: Antibody validation on spermatids and ACTL7A intracellular localization confocal analysis. (A-B) Uncropped widefield microscopy images depicting numerous WT and KO spermatids validating the absence of ACTL7A in KO mice and validating antibody specificity. (C) Confocal image of multiple WT spermatids and spermatocytes showing numerous cells exhibiting the various intranuclear and subacrosomal localization patterns for ACTL7A.

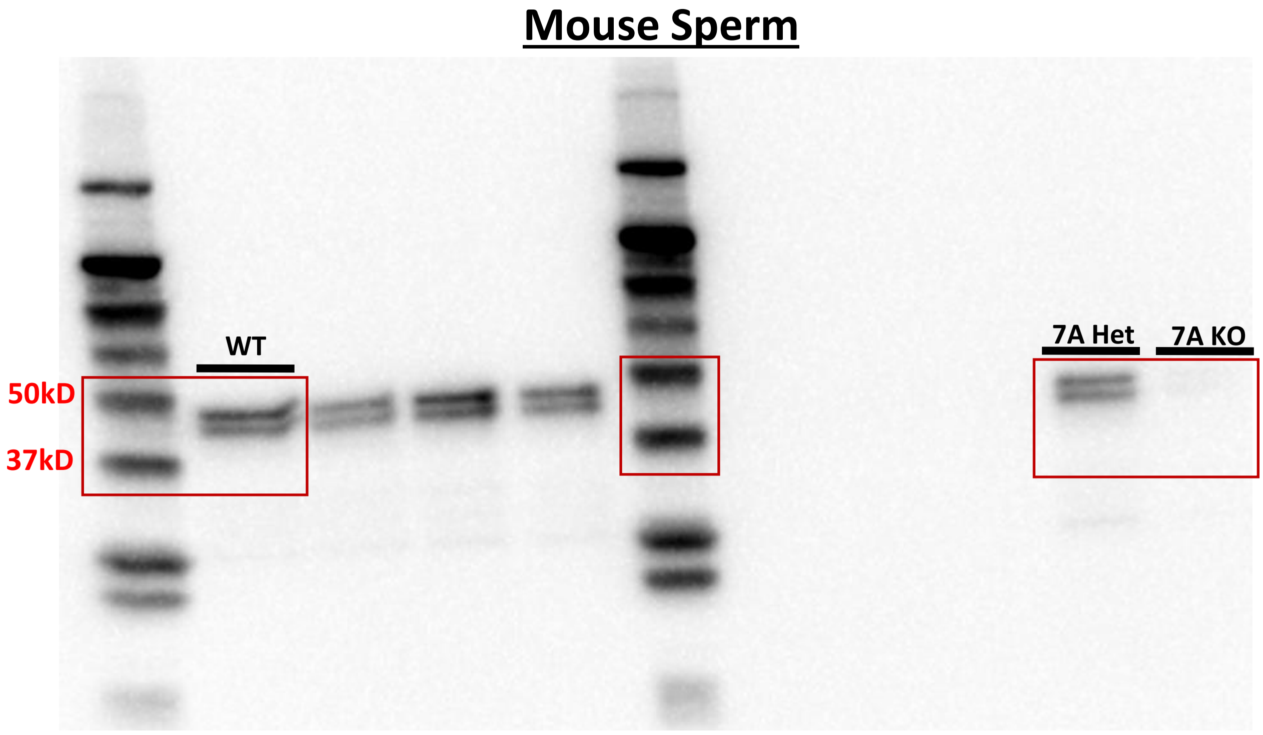

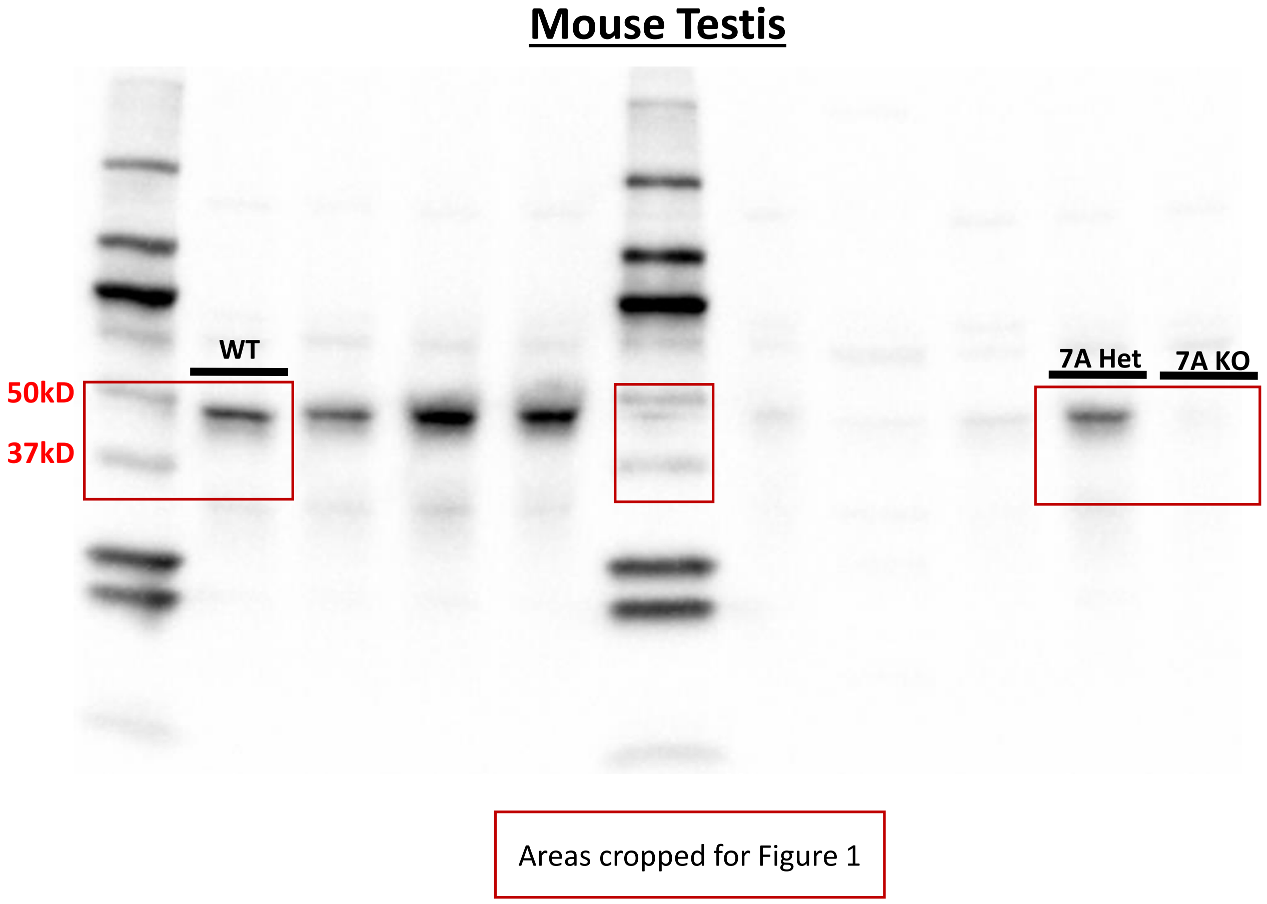

Supplementary Figure S3: Western blot products for *Actl7A* KO validation. Full western blot images of *Actl7A* KO validation shown in Figure 1.

A B

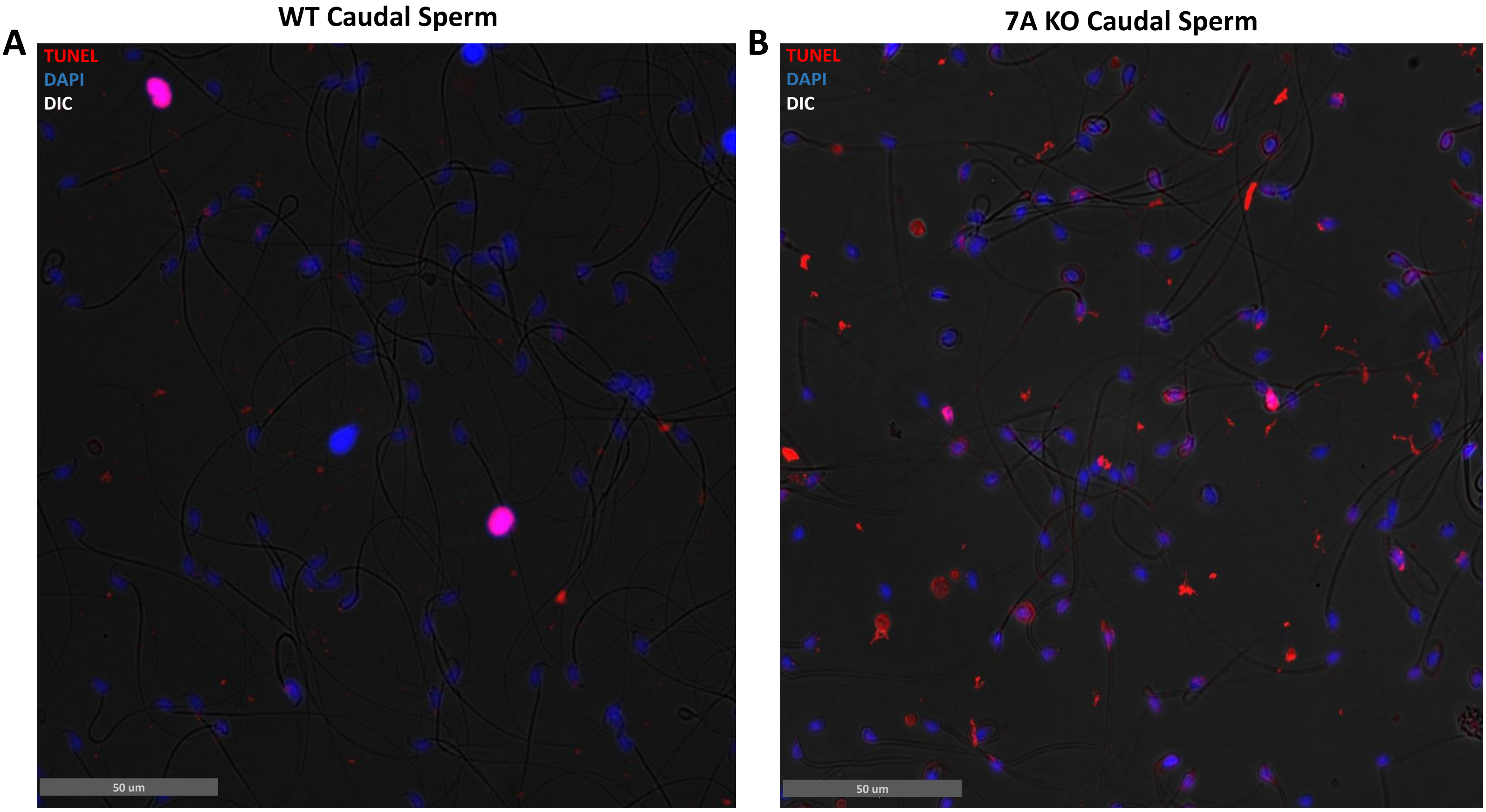

C

CASA: 15 mins

Mouse #	Static (%)	Progressive (%)	Motile (%)	Slow (%)	VCL	VAP	VSL	ALH	BCF
1630 WT	62.85	14.31	37.15	0.04	139.72	73.04	43.5	12.84	32.06
1952 WT	72.8	9.08	27.2	0	119.67	63.39	39.19	11.4	30.89
1723 WT	41.31	33.16	58.69	0	162.61	85.67	51.82	14.07	25.68
WT AVG	58.99	18.85	41.01	0.01	140.67	74.03	44.84	12.77	29.54
WT SEM	9.29	7.31	9.29	0.01	12.40	6.45	3.71	0.77	1.96
1906 7A KO	66.26	13.12	33.75	0	138.45	70.87	41.25	12.89	29.82
1910 7A KO	67.97	12.32	32.03	0	147.03	77.52	43.81	13.75	31.19
1907 7A KO	77.83	9.48	22.17	0	128.59	68.97	41.48	11.77	28.66
7A KO AVG	70.69	11.64	29.32	0.00	138.02	72.45	42.18	12.80	29.89
7A KO SEM	3.61	1.10	3.61	0.00	5.33	2.59	0.82	0.57	0.73
WT to KO T-Test (p-val)	0.3056	0.3848	0.3058	0.3739	0.8543	0.8314	0.5226	0.974	0.8765

D

CASA: 90 mins

Mouse #	Static (%)	Progressive (%)	Motile (%)	Slow (%)	VCL	VAP	VSL	ALH	BCF
1630 WT	62.21	17.4	37.8	0	136.03	75.79	47.03	12.7	29.52
1952 WT	69.05	13.71	30.95	0	134.75	75	46.58	11.9	29.49
1723 WT	50.1	26.87	49.89	0	143.04	80.96	50.74	13.12	27.87
WT AVG	60.45	19.33	39.55	0.00	137.94	77.25	48.12	12.57	28.96
WT SEM	5.54	3.92	5.54	0.00	2.58	1.87	1.32	0.36	0.55
1906 7A KO	65.11	15.42	34.89	0.1	137.39	75.77	46.53	12.84	29.44
1910 7A KO	66.23	14.37	33.77	0.04	143.24	81.73	48.17	13.85	30.33
1907 7A KO	74.48	11.72	25.52	0	125.86	67.04	42.8	11.7	31.74
7A KO AVG	68.61	13.84	31.39	0.05	135.50	74.85	45.83	12.80	30.50
7A KO SEM	2.95	1.10	2.95	0.03	5.11	4.27	1.59	0.62	0.67
WT to KO T-Test (p-val)	0.2639	0.2488	0.2637	0.1836	0.6912	0.633	0.3307	0.7709	0.1484

Supplementary Figure S4: ***Actl*7A KO sperm exhibits high levels of DNA damage and but normal motility.** (A-B) Depiction of abnormal head morphology, normal flagellar formation, and high DNA damage (red TUNEL stain) in *Actl7A* WT and KO sperm. (C-D) Computer Assisted Sperm Motility Analysis (CASA) before and after incubating sperm in BSA rich HTF media to promote capacitation. Sperm motility parameters observed appear normal between KO and WT sperm.

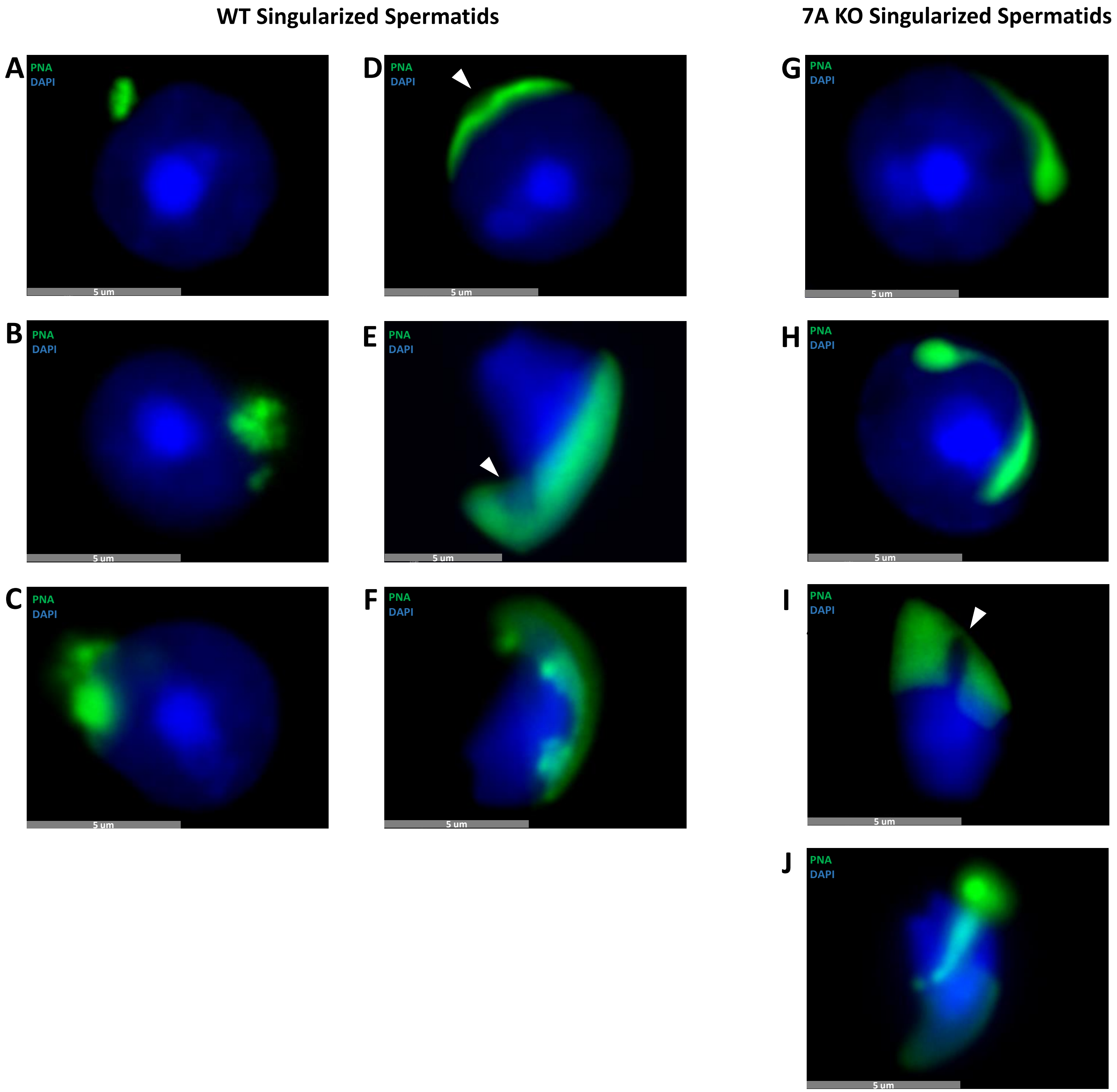

Supplementary Figure S5: Observed acrosomal defects in WT and ACTL7A KO singularized spermatids. (A) Early round spermatid showing early signs of a peeling acrosome. (B) Example of a disturbed acrosomal aggregate. (C) A fragmented and amorphous acrosomal vesicle. (D) Example of a late-stage round spermatid missing its acrosomal granule shown by the white arrowhead. (E) Irregular acrosomal distribution depicted by the lighter stained patch (arrowhead) of the acrosome near the perforatorium of an elongating spermatid. (F) Abnormal and irregular acrosomal marginal edge in a condensing spermatid. (G) Example of a lopsided acrosomal granule in an early round spermatid. (H) Late elongating spermatid with a severely displaced acrosomal granule and signs of the acrosome overly constricting the nuclear body causing aberrant morphology. (I) Elongating spermatid with a distinct piece of the acrosomal vesicle missing, depicted by a white arrowhead. (J) Condensing spermatid showing a lack of a visible acrosomal body around the perforatorium and a segment of its peeling acrosome hanging caudally to the nucleus. All images and fluorescent signals were taken in reference to healthy spermatids of each developmental stage in order to not exaggerate the aberrant acrosomal morphology and show representative images of what was considered abnormal.

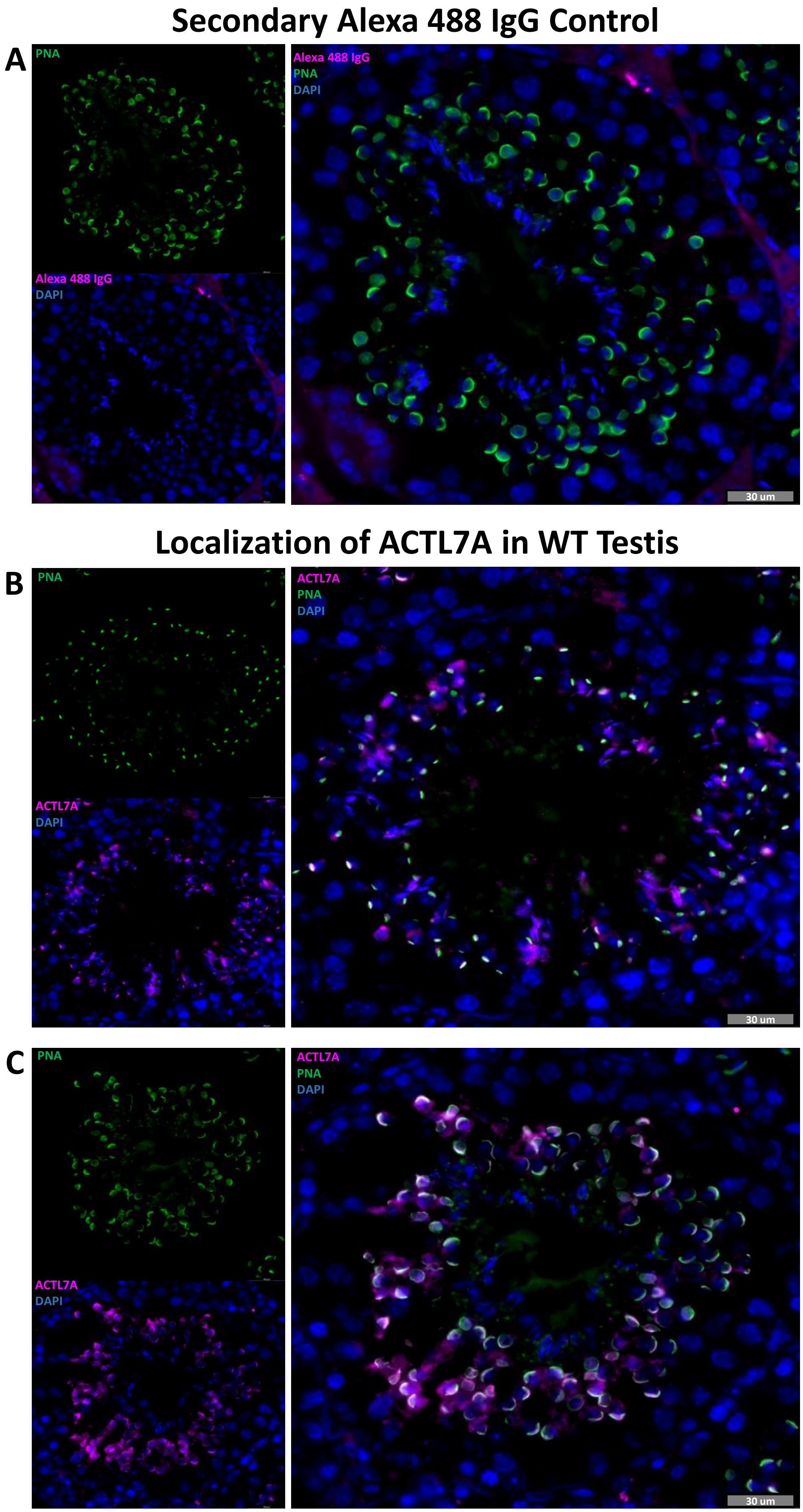

Supplementary Figure S6: ACTL7A localization by IHC is exclusively present in spermatids on WT testis sections. (A) Alexa 488 conjugated secondary control IgG localization on PFA fixed OCT-embedded testis section showing minimal to no background signal in WT tissue. (B-C) Localization of ACTL7A on PFA fixed OCT-embedded testis sections indicates exclusive localization of this protein to the germline.

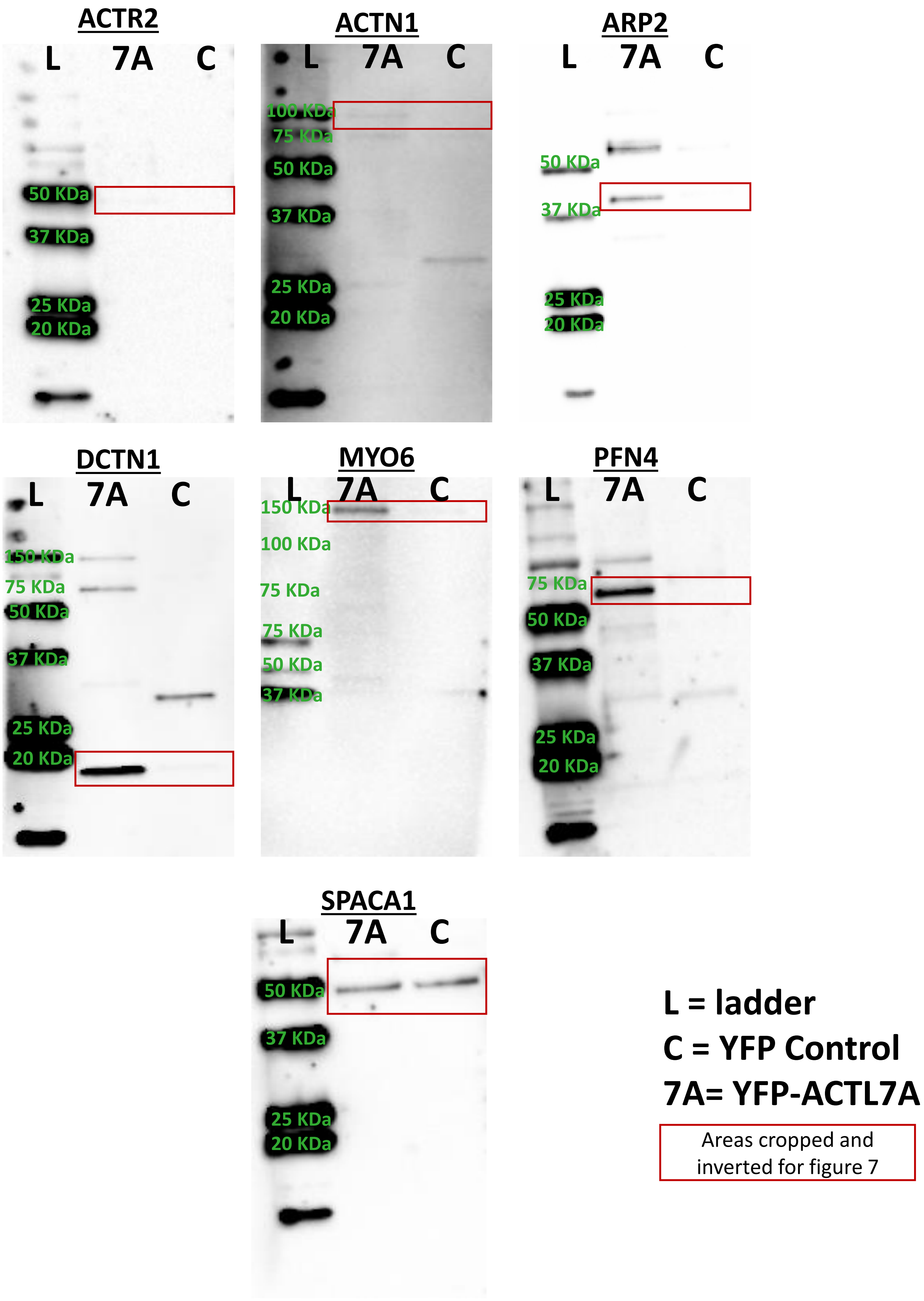

Supplementary Figure S7: Co-IP western blot products. Full Western Blot Images of Co-IP Products from the co-incubation of HEK296F expressing YFP-ACTL7A and WT testicular soluble lysates shown in Figure 7.

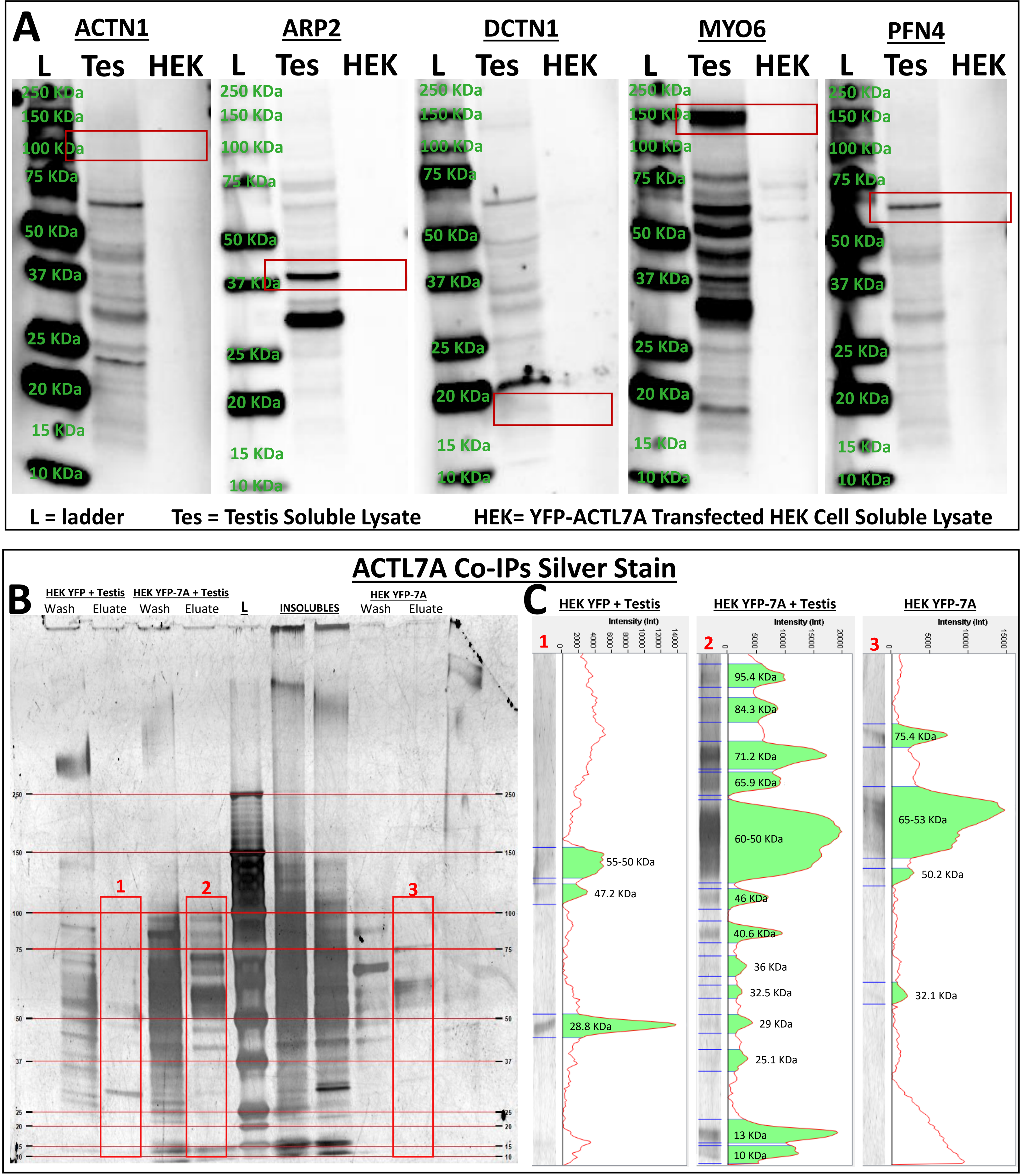

Supplementary Figure S8: Co-IP western blot Controls and Silver Stain. (A) Full Western Blot Images of Co-IP positive control (soluble testis lysate) and conditional control (YFP-ACTL7A Transfected HEK Cell Soluble Lysate) indicating the functionality of the antibodies used via the bands present in the positive control and demonstrating Co-IP product specificity by the absence of bands in the conditional control. (B) Silver stain of all Co-IP predicts and relevant washes showing total protein levels. Red boxes over blots are representative of the band sizes referenced in Figure 6. (C) Densitometry analysis of Co-IP products demonstrating the abundance and intensity of several detected molecular species below 100 KDa compared to adjacent controls.

Supplementary Table SI: Antibodies and Primers. Comprehensive list of all antibodies and primers used for this publication; key experimental parameters are indicated.

Antibodies and Labels for Histology Table

Antibody	Manufacturer	Use & Working Dilution
Alexa 488 Phalloidin	Invitrogen, # A12379	Fluorescent Microscopy (1:400)
Alexa 568 Lectin-PNA	Invitrogen, # L32458	Fluorescent Microscopy (1:400)
Alexa 488 Donkey anti-Rabbit	Invitrogen, # A21206	Fluorescent Microscopy (1:500)
HRP Goat anti-Rabbit	PerkinElmer, # NEF812001EA	Western Blot (1:5000)
Rabbit anti-ACTL7A	ProteinTech, # 17355	Western Blot (1:5000)<br>Fluorescent Microscopy (1:200)
Rabbit anti-MYO6	ProteinTech, # 26778	Western Blot (1:5000)
Rabbit anti-SPACA1	Abcam, # ab191843	Western Blot (1:5000)
Rabbit anti-ARP2	Abcam, # ab47654	Western Blot (1:5000)
Rabbit anti-ACTN1	Novus Biologicals, # NB100-92435	Western Blot (1:5000)
Rabbit anti-PFN4	MyBioSource, # MBS712699	Western Blot (1:5000)
Rabbit anti-DCTN1	Sigma, # AB6048	Western Blot (1:5000)
Rabbit anti-ACTRT2	Atlas, # HPA025079	Western Blot (1:5000)
Mouse anti-phosphorylated TYR	EMD Millipore, #05-321	Fluorescent Microscopy (1:500)
Rabit anti-Lamin A/C	Abcam, #58528	Fluorescent Microscopy (1:350)
Rabit anti-Lamin B	Abcam, #16048	Fluorescent Microscopy (1:350)

Primers used Table

Primer	94°C 2min → (94°C 30sec , xx°C 30sec , 72°C 30sec) x 40 → 72°C 3min → 16°C ∞			
Strain			annealing Temp.	PCR size
Actl7a	KO : Primer Fw (5' → 3')	ACCAGATAAGGGTGGGGTTC		
	KO : Primer Rv (5' → 3')	TTGCTCCCGTGAGAGACTTT	60°C	596 bp
	WT : Primer Fw (5' → 3')	ACCAGATAAGGGTGGGGTTC		
	WT : Primer Rv (5' → 3')	GTGCCCCACAAAGGTCTCT	60°C	675 bp
